# Calorimetric Investigation of the Relaxation Phenomena in Amorphous Lyophilized Solids

**DOI:** 10.3390/pharmaceutics13101735

**Published:** 2021-10-19

**Authors:** Sebastian Groёl, Tim Menzen, Gerhard Winter

**Affiliations:** 1 Department of Pharmacy, Ludwig-Maximilians University Munich, 81377 Munich, Germany; 2 Coriolis Pharma Research GmbH, 82152 Munich, Germany; tim.menzen@coriolis-pharma.com

**Keywords:** collapse, controlled nucleation, differential scanning calorimetry (DSC), freeze-drying, isothermal microcalorimetry (IMC), lyophilization, molecular mobility, relaxation

## Abstract

Studying the thermal history and relaxation of solid amorphous drug product matrices by calorimetry is a well-known approach, particularly in the context of correlating the matrix parameters with the long-term stability of freeze-dried protein drug products. Such calorimetric investigations are even more relevant today, as the application of new process techniques in freeze-drying (which strongly influence the thermal history of the products) has recently gained more interest. To revive the application of calorimetric methods, the widely scattered knowledge on this matter is condensed into a review and completed with new experimental data. The calorimetric methods are applied to recent techniques in lyophilization, such as controlled nucleation and aggressive/collapse drying. Phenomena such as pre-*T_g_* events in differential scanning calorimetry and aging shoulders in isothermal microcalorimetry are critically reviewed and supplemented with data of freeze-dried products that have not been characterized with these methods before.

## 1. Introduction

In the pharmaceutical field, there is still a tremendous need for lyophilized products with increasing tendency. More and more biological compounds are being developed and used to improve the treatment of severe diseases [1]. The advantages of biotec products are obvious. Monoclonal antibodies, for instance, provide improved targeting upon conjugation with small-molecule drugs with low side effects for specific cancer treatments [2]. Protein- or RNA-based vaccines allow us to react quickly to pandemic infections, as we have experienced recently. These are only a few of the manifold reasons to use these products [3,4,5]. However, many biological drug products suffer from physico-chemical instabilities, which compromise their long-term stability [6]. Several instabilities, such as oxidation, hydrolysis, or aggregation, make it difficult to develop a stable pharmaceutical formulation that can be shipped, stored, and administered conveniently [7,8,9,10,11]. Therefore, liquid biological drug products typically require long-term storage under frozen or refrigerated temperatures. In contrast, freeze-drying as a gentle method to remove the water provides an opportunity for the storage of freeze-dried drug products under ambient conditions by overcoming most of physico-chemical instabilities [12,13]. The resulting dry solid matrix is able to protect the biological ingredients, although most of the water, which is the natural habitat of these compounds, is missing [13]. This is not trivial and only a few drying methods are able to provide a stabilizing matrix [14,15]. The secret of these methods is hidden in the special properties of the resulting matrix, which will be explained in the following.

A typical freeze-drying process leads to a fully amorphous, glassy system or at least a partly amorphous one [16]. Several studies proved that biologicals such as proteins are much better preserved in the amorphous state compared to a crystalline one [17,18]. There are two main theories that try to explain why this is the case. One is the water replacement theory that states that biologicals are best preserved in their native state, which is surrounded by water [19]. When the water is removed during the freeze-drying process, excipients such as sugars stabilize this native structure by replacing the water with their own hydroxyl groups [19,20]. This can only work when the excipient is in an amorphous state. The other approach is the glassy dynamics theory, also called the vitrification hypothesis [19,21]. Summarized, it states that the matrix becomes more and more rigid during its formation in such a manner that any movement of molecules and functional groups is finally impossible. In this context, movement is seen as an essential factor for reaction processes that are impeded in this way [22,23]. This theory is the major basis of the topics discussed in this review and thus is further explained in the Theoretical Background section.

Importantly, these amorphous products are thermodynamically instable or metastable, which is, on the one hand, helpful due to the improved solubility of the formulations but is a problem for long-term stability [17]. Thus, the formulations will rearrange their structure, which is called structural relaxation, or even crystallize during storage, thereby at least partly losing their ability to stabilize the biological entity [24]. This is also relevant for small drug molecules. The reason to prepare an amorphous form is often not only to increase water solubility but to achieve an enabling rise in bioavailability [25,26,27]. Changes in this amorphous state over time or even crystallization can lead to critical dose fluctuations [28]. For this reason, it is in fact mandatory to analyze the solid state with respect to the stability predicting factors. Most common solid state properties in this context are residual moisture, specific surface area, glass transition temperature, and crystallinity. Furthermore, one of the more recent techniques is the investigation of the ability of a lyophilized molecule to exchange hydrogen atoms with deuterium (solid state hydrogen deuterium exchange, ssHDX), thereby indicating the accessibility and reaction rate of degradation or—in other words—the ability of a matrix to provide immobilization to the molecule that shall be stabilized [29]. All these methods and properties are indeed very important for the characterization of a freeze-dried product but do not directly mirror (except the glass transition temperature) the thermal history of the lyophilized matrix, which is strongly connected to the thermodynamic properties and thus the long-term stability of freeze-dried products.

Different researchers agree that relaxation processes in freshly prepared glasses (amorphous excipient/drug matrices) can be used as a good surrogate to characterize their thermal history and thermodynamic properties and can also be taken as a predictive tool for the long-term stability of the created products [30,31]. Several methods were used and developed in the last few decades to investigate matrix relaxations. These include, inter alia, measurements of viscosity [16,32], solid state nuclear magnetic resonance spectroscopy (ss-NMR) [22,33], dielectric spectroscopy [16,22,34], terahertz spectroscopy [22,35], thermally stimulated depolarization current [36,37], electron spin resonance spectroscopy [38], incoherent neutron scattering [39,40] and, last but not least, calorimetric methods [17,22,41,42]. With the exception of the latter, all these methods require very well-trained scientific operators and a great deal of experience to obtain and evaluate the relaxation data. Furthermore, most laboratories that deal with pharmaceutical freeze-drying have only limited access to these methods. In contrast, almost every formulation laboratory has access to at least one calorimetric method—typically differential scanning calorimetry (DSC)—but only a few scientists make use of the full potential of this device.

With this review, we wish to offer a summary of the thermal history and relaxation determination of lyophilized pharmaceutical products with calorimetric methods. We hope to encourage the reader to “revive” the partly neglected and surprisingly simple techniques and re-evaluate their use, particularly regarding the recent updates in freeze-drying techniques. We provide a guideline on calorimetric methods and encourage researchers to acquire more information from calorimetric measurements. We complete this review with our own data and look at the calorimetric methods from a different angle in the context of collapse freeze-drying, controlled nucleation, and highly concentrated protein formulations.

## 2. Theoretical Background

A thermodynamic equilibrated state can be described as a system where every molecule or functional group possesses its full degree of freedom [43]. This means, for example, that the system adapts to changes in temperature with modifications in its structure or other thermodynamic properties such as configurational entropy immediately [23,43]. This system is called “ergodic” [44]. Examples of thermodynamic equilibrated material are crystalized solids and liquids, even when the latter are supercooled (exceptions may exist).

**Figure 1:**
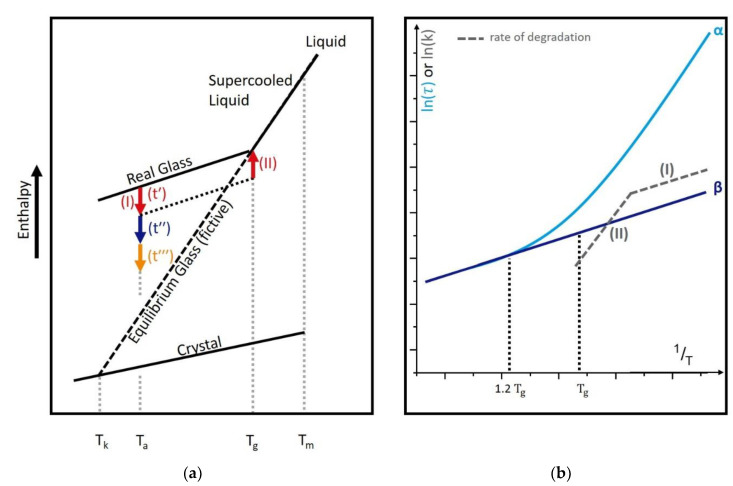
Theoretical considerations concerning relaxation. (**a**) Enthalpy relaxation diagram of solids with the melting temperature $T_{m}$, the glass transision temperature $T_{g}$, the annealing or tempering temperature $T_{a}$, and the Kauzman temperature $T_{K}$. (t′)–(t‴) symbols indicate the tempering process of a glass at a certain temperature $T_{a}$. In the context of this review, the “real glass” represents our freshly prepared lyophilized formulation. The diagram is modified and adapted to the focus of this review [16,17,23,43,44,46,48]. (**b**) Model of the dominant relaxation modi. The influence of α- and β-relaxations on a degradation process with the constant k in dependence on temperature and $T_{g}$. (I) degradation is governed by β-mobility; (II) degradation is governed by α-mobility [43].

Usually, when a liquid is cooled, at a certain point, the melting temperature $\left(T_{m}\right)$, the liquid crystallizes and forms a solid [23]. During the freezing step of a lyophilization process, the solution normally fails to fully crystallize. In detail, parts of the water will crystallize, leading to an up-concentration of the remaining solution. This solution, containing the active pharmaceutical ingredient (API) and the excipients, will thereby form a supercooled liquid [23]. With further cooling of this supercooled liquid, at a certain point, the glass transition temperature $\left(T_{g}\right)$, the system becomes too slow to follow the cooling rate with thermodynamic changes. Thus, it leaves the equilibrated state and the material forms a glass. Concerning lyophilizates, it is important to bear in mind that we can essentially identify two glassy systems. One glass system is the solution before drying, with $T_{g}$ values of around −30 °C, also called $T_{g}^{\prime }$, and the other system after drying, with only little remaining water content. Depending on the excipients, the $T_{g}$ can be at temperatures far above 100 °C. Important is that also the latter system is called a supercooled liquid, from the thermodynamic perspective, when it is heated above its $T_{g}$. Although the system can be considered a solid after freeze-drying, it is not in a thermodynamic equilibrium below $T_{g}$. Thus, the fresh glass will have an excess in entropy, enthalpy, and volume [23,31,44]. In Figure 1a, an energetic model is provided that illustrates these explanations. The dashed line is a fictive, non-existent state that describes the thermodynamic equilibrated state of the system. Relaxation means the process of the new material approaching the “equilibrated glassy state”, which can be considered the prolonged energy state of the supercooled liquid (dashed line) [23]. This process, also called the physical aging of a glass, is connected to changes in other material properties, which strongly influences the protein stability [31,45]. The energy excess released below $T_{g}$ during the aging provides the required energy for molecular movement and degradation [41,46]. Slowing down this processes hence has the potential to increase the stability of lyophilized products [47]. Therefore, an understanding and optimization of the thermodynamic properties of the host glass is essential to improve the long-term stability of a pharmaceutical formulation [47].

### 2.1. Nomenclature of Relaxation Modi

The relaxation processes themselves are classified into different modi concerning their behavior in a broadband relaxation spectroscopy measurement. They are sorted into *α*-, *β*-, and *γ*-relaxations [22,46,49], with *α*-relaxations being the slowest relaxations (time scale from seconds to months), followed by *β*-relaxations (time scales of milli- to picoseconds) and *γ*-relaxations (beyond *β*-relaxations in the time scale of picoseconds) [22,43,46,50]. Apart from a limited number of exceptions, calorimetric methods capture only *α*-relaxations [51,52]. Thus, this review focuses on the properties of *α*-relaxations and refers to other excellent publications for more information on other types of relaxation modi [46,50,53]. For the investigation of further relaxation modi, other methods are appropriate, such as inelastic neutron scattering (β-relaxations), ss-NMR (β-relaxations), and dielectric spectroscopy (β- and γ-relaxations) [22,46].

*α*-Relaxations, also known as “structural relaxations”, are strongly temperature-dependent and mostly linked to viscosity [23,46,47,50]. Thus, they describe the translational and rotational movement of whole molecules [22,23,31,54]. One well-known parameter that is connected to *α*-relaxations is the glass transition temperature and so it was a long-used idea to simply increase the temperature of the $T_{g}$ of a formulation to slow down processes related to *α*-relaxations [47,55]. On the one hand, it is proven in several studies that *α*-relaxations as well as the $T_{g}$ contribute to physical stability, but, on the other hand, for single amorphous drugs, sometimes, *β*-relaxations seem to be a better predictive tool [22,46,47,56]. Luthra and Cicerone developed a model that is able to explain this fact [43]. Whereas β-relaxations are temperature-independent on a wide temperature scale and thus always contribute the same amount of energy to mobility, the influence of α-relaxations strongly increases near $T_{g}$ and dominates the glassy dynamics in this temperature range (Figure 1b) [43]. The exact intersection temperature varies between formulations and this leads to different scenarios about whether *α*- or β-relaxations are the best stability predictors in a certain range of $T_{g}-T_{a}$ [43].

As mentioned above, not many recent studies exist that use the calorimetric methods in the context of new process developments in freeze-drying, although it is emphasized in the community that the properties of a glass depend on the “history” (process) of how it was made [46,57]. Below, the theoretical considerations as well as the evaluation of *α*-relaxation processes of freeze-dried products with DSC and isothermal microcalorimetry (IMC) are described in detail, including the relevant thermodynamic terms and formulas.

### 2.2. DSC

Firstly, it is of the utmost importance to point out that not all investigated parameters in glasses are thermodynamic parameters but rather of a kinetic nature [46]. This is easier to understand if the explanation above is combined with the practical example of the DSC measurement. For a measurement, normally, 5–10 mg of sample is hermetically sealed in an aluminum pan. The pan is then placed in the oven of the DSC instrument next to an empty reference pan. During a fixed heating ramp, the heatflow of the sample is compared to the reference, which makes it possible to detect thermal events that trigger changes in the heatflow. In the pharmaceutical environment, usually, heat ramps from 1 K/min up to 20 K/min [58,59,60] are used. Bearing in mind that “at a certain point the system becomes too slow to follow the cooling rate with thermodynamic changes” helps to explain the kinetic character. This “certain” point depends inter alia on the heating rate of the DSC. General advice and developments in DSC measurement methods are perfectly described elsewhere [58,61]. In the following, we wish to emphasize details that play a role in relaxation and molecular mobility and are often found in the non-pharmaceutical literature.

**Figure 2:**
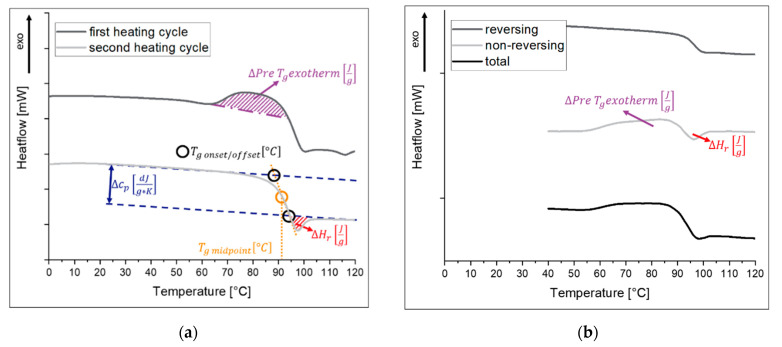
DSC thermograms of a freeze-dried trehalose-based pharmaceutical placebo formulation. The curves are displayed with offset for a better graphical presentation: (**a**) measured with a linear temperature program with two heating cycles; (**b**) measured in modulated mode (mDSC).

In nearly every DSC curve of a lyophilized sample, proof of sample aging or, rather, energy loss due to relaxation is observable but barely recognized. Figure 2 shows different thermograms of a freeze-dried trehalose-based pharmaceutical placebo formulation. In Figure 2a, a linear measurement mode is displayed. The following thermodynamic parameters can be evaluated:

$∆c_{p}$*and*$T_{g}$*, Figure 2a blue arrow and yellow circle, reversible.* These are the “standard” parameters to investigate freeze-dried formulations and thus are perfectly described elsewhere [62,63,64,65]. However, the authors wish to point out a few aspects.

At first, the $T_{g}$ is strongly dependent on the heating rate and on the residual moisture of the formulation. Due to the fact that water works as a plasticizer, the rule of thumb is that per 1% more residual moisture, the $T_{g}$ decreases by around 10 K [66].

Secondly, to pierce or not to pierce the crucible? In some publications, it is recommended to pierce the lid of the sealed aluminum pan. There is no right or wrong but the following points have to be considered:

- At temperatures higher than 60 °C, residual moisture will evaporate out of the measured matrix, which lowers the $T_{g}$ significantly and finally delivers data for the thermal behavior of a dry sample. However, this is not the property of the samples that is stored in the product vials after freeze-drying.
- In the context of the thermal history of an amorphous solid, the value of $∆c_{p}$ at $T_{g}$ is relevant. It is the comparison of the $c_{p}$ (“baseline”) between pre- and post-$T_{g}$ and can be determined as shown in Figure 2a. It must not be confused with the $c_{p}$ value itself, which is a substance constant and declares the needed heat to raise 1 g of sample by 1 K. With a pierced lid, the values are determined isobaric in contrast to a hermetically sealed pan. Moreover, it has to be kept in mind that$\;∆c_{p}$ at $T_{g}$ depends on the absolute sample weight. Thus, in multiple measurements (e.g., triplicate), the values of $∆c_{p}$ will differ more strongly than the $T_{g}$ temperature due to additional weighing errors. The results of $T_{g}$ are quite mass-independent.

*Enthalpy recovery*$∆H_{r}$*, Figure 2a red area, non-reversible.* This is also called $T_{g}$-overshoot and is the area that prolongs the post-$T_{g}$ baseline [22]. It represents the weight-normalized restored enthalpy that a sample releases during storage at temperatures below $T_{g}$. This storage below $T_{g}$ is also called annealing, aging, or tempering depending on the literature [22,67]. Throughout this review, tempering is used because it does not interfere with the name of certain process steps in a lyophilization process. The released enthalpy arises from the energy excess that an amorphous sample possesses in comparison to the thermodynamic equilibrated state (e.g., crystalline form). During storage below $T_{g}$, this energy excess is slowly released from the sample. This value is needed later for determining relaxation times with DSC.

∆*Pre-*$T_{g}$*exo- or endothermal events, Figure 2a magenta area, non-reversible.* This event is rarely reported and is the most unclear observation in the DSC analysis of lyophilizates [43,68]. Dependent on the formulation, it can be sharp (e.g., sucrose) or broad (e.g., trehalose) as well as endothermic (e.g., sucrose) or exothermic (e.g., trehalose) [43]. Moreover, it is reported for pure protein formulations (lyophilized bovine serum albumin, BSA), where it is explained as “*α*-relaxation-like protein transition” and termed the “protein dynamical temperature” [68]. Because this event occurs in lyophilizates but not in amorphous melt-quenches, it is suggested that this peak arises from the special production procedure of a freeze-dried sample being frozen in an open structure with subsequent water removal [43,67,69,70]. One could describe the process in a way that the freezing step produces a glass, which is then converted into another glass by water sublimation. Because this pre-$T_{g}$ event can be sharpened by sample tempering, it could be seen as a proof for widely distributed relaxation states that can be condensed during longer storage times [43,58,69]. The relaxation state theory is reasonable for a pre-$T_{g}$ exotherm, but not for a pre-$T_{g}$ endotherm. In this case, Luthra suggested that such samples are accidently tempered during the secondary drying step of the freeze-drying process, thereby losing their energy excess [43]. As a result, an endotherm occurs because the system receives enough energy to recover the lost energy excess at this point [43,71]. This is in agreement with Vyazovkin and Dranca, who investigated *β*-relaxations with a DSC method [52]. They stated that the endothermal peak occurs immediately above the annealing temperature and is a sign that *α*- and *β*-relaxations are coupled around the glass transition region [52]. Further, this would support the model of dominant relaxation modi of Luthra and Cicerone (Figure 1b) [43]. In contrast, Reddy et al. oppose this idea in their studies comparing DSC and thermally stimulated current (TSC) by naming events “*β*-relaxations” when observed in the TSC but not visible in DSC [72]. Moreover, Wang et al. were not able to correlate these pre-$T_{g}$ events with NMR- and neutron backscattering-measured β-relaxations [70]. On the contrary, these TSC events can be found in IMC measurements and it is suggested that IMC is able to measure some kinds of β-motions as well as more detailed α-motions [37]. For polymers, four properties of this pre-$T_{g}$ phenomenon are reported; the effect is asymmetrical, the endotherm increases when the tempering time or temperature is raised, it can be superimposed from the glass transition, and the temperature peak is a function of tempering time ($\ln{\left(t\right)}_{tempering}$) and temperature (${\left(T\right)}_{tempering}$) [73,74]. In the event that the pre-$T_{g}$ event is missing, it is suggested that it is superimposed by the glass transition, as described previously [74]. It should be pointed out that this pre-$T_{g}$ event is not based on a particular interaction between the protein and the placebo matrix because it occurs in placebo formulations as well as in pure freeze-dried protein and thus is a thermodynamic phenomenon (see also Thiewes and Steeneken [74]). Once the thermal history of a sample is erased by heating above $T_{g}$, this pre-$T_{g}$ event disappears. The remarkable fact that the samples with missing pre-$T_{g}$ events are often from quench-cooled melts and not from freeze-drying processes emphasizes the value of using calorimetric matrix investigations of lyophilizates. It is possible to distinguish between different production methods of the sample by a single measurement and such observations make clear how strongly the process influences the sample properties.

If there is no interest in investigating the thermal history of a sample and only the $T_{g}$ temperature is needed, it would be recommended to perform two heating cycles of the product with a $T_{g}$ evaluation of the second heating cycle (Figure 2a, second heating cycle). The potentially interfering influences of the matrix effects such as the pre-$T_{g}$ exotherm throughout the thermal history of the sample would then be eliminated. This can clearly be seen in Figure 2a. Even the enthalpy recovery ($∆H_{r}$) could be removed if the sample is held at a temperature above $T_{g}$ for a longer time, with subsequent rapid quench cooling. For this, a cooling rate of 20 K/min or higher is suggested [61]. With this, a quasi-fresh glass is produced in situ that is “free of any thermal history”. However, it has to be ensured that no irreversible changes such as crystallization or decomposition happen when the sample is heated above $T_{g}$.

An even better way to capture all the thermal events of a sample would be DSC in the modulated mode (mDSC) [75,76]. With this method, the heat signals can be separated in reversing (e.g., $T_{g})$ and non-reversing signals (e.g., melting temperature, crystallization, $∆H_{r}$, pre-$T_{g}$ event). Further explanation of the methology can be found, e.g., in [77,78]. As seen in Figure 2b, with one measurement, all described thermal events can be easily found and, in contrast to the linear mode, even superimposed events can be distinguished.

*Determination of relaxation times with DSC, an indirect method.* Figure 1a shows, aside from crystallization and melting, practically all DSC events of an amorphous pharmaceutical sample. During an α-relaxation process, the relaxation energy is released, which is considered a certain part of the amorphous sample compared to the thermodynamic equilibrated state. To determine α-relaxation times with this DSC signal, an assumption has to be made. The relaxation energy itself, the value of interest (Figure 1a, pathway (I)), is equal to the enthalpy recovery at $T_{g}$ (Figure 1a, pathway (II)). This enthalpy recovery corresponds to the red area in Figure 1a and the red marked peak in Figure 1b, $dH_{r}$. To point out the consequences for the practical (quite laborious) measurement: to obtain the complete relaxation curve at the temperature of interest (*T*), several DSC pans of the formulation that should be investigated have to be prepared. Afterwards, each pan must be stored at the same temperature (*T*) for a different amount of time (*t*) and then heated above the glass transition temperature. The value of $∆H_{r}\left(t\right)$ can be estimated in this way and the value should increase with increasing storage time (*t*) of the sample. At least one pan is needed for each point of $∆H_{r}\left(t\right)$. This enthalpy recovery energy at time (*t*) can then be used to calculate the corresponding point in the relaxation curve. The theoretical considerations are described in the following.

Regularly, *α*-relaxations in pharmaceutical glasses follow a non-exponential decay. This arrives from independently relaxing substates of different size and distribution [43]. These substates can be imagined as different locations in the sample with individual energy excess. They might be compared (not connected) with the distribution of single compounds in a mixture. Mathematically, the overall relaxation process of a sample as a whole can be described best with the non-exponential decay function suggested by Kohlrausch–Williams–Watt (KWW), also called the stretched exponential function [48,79,80,81].

(1) $$\;\phi_{KWW}\left(t,T\right)=exp\left[{\left(-\frac{t}{\tau \left(T\right)}\right)}^{\beta }\right]\;$$

In approximation, τ can be imagined as the relaxation time of each single relaxation step (in hours) and β as a distribution of the relaxation states [22,23,82]. β can only have values between 0 and 1, which should be used as limiting borders for the non-linear curve fitting [17,22,23]. The closer the values of β to 1, the more homogeneous is the distribution of relaxation states [53]. This means that the described substates of the whole sample possess a quite similar energy excess. Due to the fact that the KWW equation is an empirical equation, other interpretations are possible [23]. To obtain the single data points of the KWW equation, the obtained $∆H_{r}\left(t\right)$ as described above has to be converted by the following formula.

(2) $$\phi_{KWW}\left(t,T\right)=1-\frac{∆H_{r}\left(t\right)}{∆H_{r}\left(\infty \right)}\;$$

In this equation, $∆H_{r}\left(t\right)$ was already explained in Figure 2a (red area) and $∆H_{r}\left(\infty \right)$ is the infinite relaxation. The latter is the theoretically possible maximum enthalpy recovery at infinite time [17]. This maximum enthalpy recovery could be represented by one of the DSC pans described above that age over several years. They have been aged for so long (infinite) that the energy recovery will not increase anymore, even with a longer aging time (*t*). Because this would not work in practical experiments, Formula (3) is used to determine the value of $∆H_{r}\left(\infty \right)$.

(3) $$∆H_{r}\left(\infty \right)=\left(T_{g}-T\right)\times ∆c_{p}\;$$

T is the temperature of interest for which we wish to determine the relaxation times. $T_{g}$ and $∆c_{p}$ are the glass transition temperature and the heat capacity change at the glass transition temperature, respectively. More simply, the idea behind Formula (3) is that, at temperatures above $T_{g}$, the thermodynamics of our sample correspond to a supercooled liquid. As already described, the $T_{g}$ is the point during cooling where a supercooled liquid falls out of equilibrium and forms a glass. Thus, reversing this step, heating above $T_{g}$ brings the formulation back to thermodynamic equilibrium. As a result, all energy excess is released at $T_{g}$ and is measurable as $∆c_{p}$ at this point. Thus, $∆c_{p}$ at $T_{g}$ (Figure 2a blue description) makes it possible to determine the maximum energy release that is possible during the storage time even below $T_{g}$ at a certain temperature. As seen in Figure 1a, the discrepancy between the equilibrated state (dotted line) and the glass increases with growing distance to $T_{g}$. Therefore, Formula (3) simplifies the temperature dependence in $∆c_{p}$ and is considered state of the art for the determination of $∆H_{r}\left(\infty \right)$ [23,83].

To summarize the analytical process, $∆H_{r}$ values are measured for different times (*t*) at the temperature of interest (*T*) and then converted into data points for the relaxation curve using Formula (2). The relaxation curve itself can then be drawn and fitted with Formula (1) to obtain the relaxation time constants τ and β.

For a more detailed understanding of how the formulas are derived mathematically, the following literature is referred to [17,22,23,43,53,84]. Formula (4) condenses the verbalized process into a concluding mathematical relation.

(4) $$exp\left[{\left(-\frac{t}{\tau \left(T\right)}\right)}^{\beta }\right]=\phi_{KWW}\left(t,T\right)=1-\frac{∆H_{r}\left(t\right)}{∆H_{r}\left(\infty \right)}\;$$

To clarify, with this procedure, the relaxation times are measured indirectly. This involves interrupting the relaxation process at a time (*t*) and measuring to what extent the sample is already relaxed compared to its maximum relaxation ($∆H_{r}\left(\infty \right)$). This approach neither shows the real rate of the relaxation process (what happens in between the obtained data points) nor measures the relaxation with certainty due to possible side reactions in the DSC measurements that increase or decrease $∆H_{r}$. Due to these artificial side reactions, $∆H_{r}$, and thus the relaxation energy, are wrongly determined to be higher or lower than their actual value.

With the described procedure, the investigation of relaxation time constants τ and β with DSC applying the KWW function corresponds to a kinetic model. It uses $∆H_{r}$ as a function of aging time and thus assumes that the relaxation time τ of a sample is independent of the measurement time (*t*) [43]. However, the relaxation time τ is actually not a constant and will increase during the aging time [43]. This can be explained quite logically because, over time (*t*), the formulation approaches its equilibrated state and the energy gradient between the energy excess and the equilibrated state minimizes. Thus, the relaxation process decelerates and the relaxation times rise. In scientific terms, the fictional temperature $T_{f}$ decreases during storage below $T_{g}$ [85].

Nevertheless, practically, this method works well, although a few theoretical simplifications have to be made. The determination of relaxation times could be improved by a direct measurement of the relaxation rate, which is not possible with DSC but with IMC, which is explained in the next section.

### 2.3. Isothermal Microcalorimetry (IMC), Direct Measurement of Relaxation

**Figure 3:**
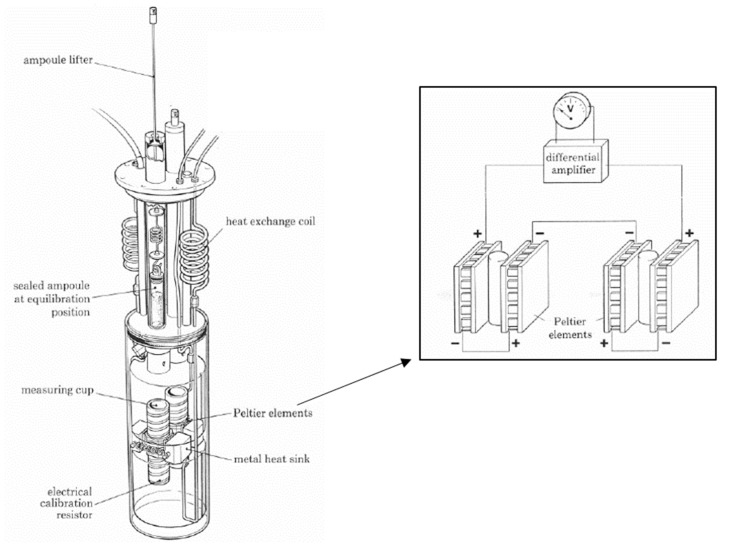
Measurement cylinder of the isothermal microcalorimeter. The small picture shows a magnified view of the Peltier elements. With courtesy from TA Instruments [88].

The difficulty in the direct measurement of *α*-relaxations is the long time scale of the reaction, which can be up to several months, and also the non-ergodic behavior of the system [16,47]. However, as mentioned before, *α*-relaxations are dependent on more factors than simply the storage temperature and $T_{g}$ and thus should be measured directly [47]. To make this possible, a very sensitive instrument with a high resolution and baseline stability is needed, which a DSC cannot provide. An isothermal microcalorimeter is capable of providing these features as it was originally developed to investigate microbiological reactions with very low and latent heat [86,87]. In principle, this instrument works similarly to a DSC. The sample is transferred to a measurement ampoule, which is then, together with a reference ampoule, inserted at the equilibration position of the instrument (Figure 3). This position is needed to bring the sample to the measurement temperature. Due to the high sensitivity of the instrument, direct insertion into the measuring cup would lead to a signal overshoot connected to disturbing noise. Thus, the sample is held at an elevated position for 10–60 min before it is lowered into the measuring position.

**Figure 4:**
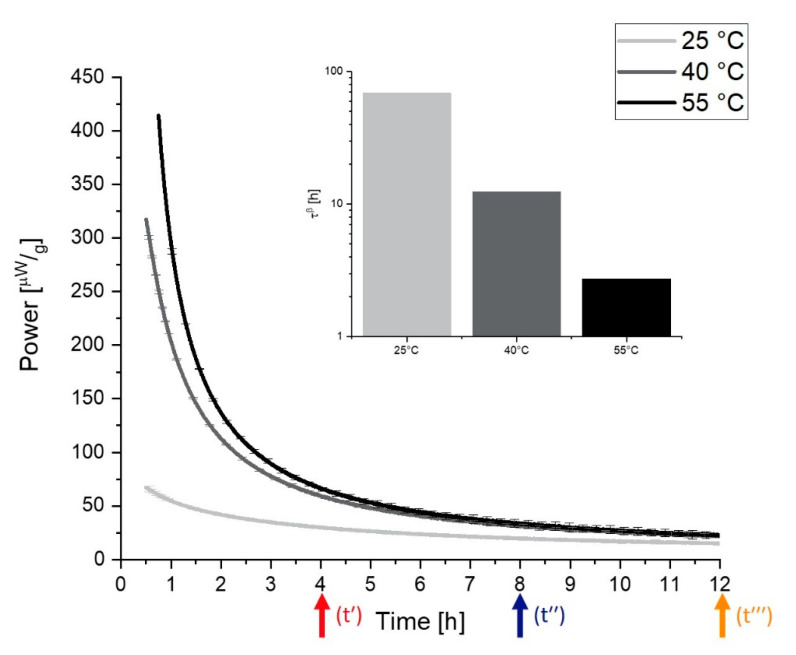
Relaxation curves of the same trehalose-based formulation as in Figure 2 obtained by isothermal microcalorimetry at different measurement temperatures. The small diagram represents the respective calculated $\tau^{\beta }$ values. The markers (t′)–(t‴) represent the different relaxation states of the formulation compared to Figure 1a as a fictive example.

The measuring cups are connected with Peltier elements that measure the differential heat signal between the measurement and reference ampoule. In contrast to a DSC, the IMC only takes isothermal measurements at one defined temperature, but the stability of this temperature is much higher than using a DSC in an isothermal mode. This is guaranteed with a large heat sink—in practice, a 25 L water (or oil) bath that controls the temperature within 0.0001 K [86,88]. Thus, the general noise of an IMC can be reduced to ±10 nW in the short term and to a baseline stability to $\pm 40\frac{nW}{24\;h}$. As demonstrated in Figure 4, an isothermal measurement at 25 °C results in a normalized maximum heat signal of approximately 75 $\frac{µW}{g}$ for the objective to measure relaxations in freeze-dried products. With a sample weight of around 300 mg, this means an absolute signal of 22.5 $\frac{µW}{g}$. A DSC with a baseline flatness of ±100 µW would not be able to separate such a relaxation signal from noise [87].

Thus, IMC is able to measure the rate of a relaxation $\left(\frac{∆H_{r}\left(t\right)}{∆t}\right)$ reaction directly during the tempering process, in contrast to the method with DSC [17]. For this reason, the KWW function in Formula (1) has to be differentiated to bring the power output of the IMC measurement in connection with a relaxation enthalpy (mathematically, power is the derivative of energy).

(5) $$P=277.8\times ∆H_{r}\left(\infty \right)\times \frac{\beta }{\tau }\times {\left(\frac{t}{\tau }\right)}^{\beta -1}\times exp\left[-{\left(\frac{t}{\tau }\right)}^{\beta }\right]\;$$

Formula (5) above presents the differentiation of the KWW function with the number 277.8 being a result of conversion of units and P the normalized power in $\frac{\mu W}{g}$ [17]. This formula has to be improved to avoid errors in the evaluation near t=0 due to the fact that the power P approaches infinity when time approaches zero [17]. For this reason, the formula postulated by Peyron et al. was adopted for the evaluation of IMC data by Liu et al. [17,89].

(6) $$P=277.8\times \frac{∆H_{r}\left(\infty \right)}{\tau_{0}}\times \left(1+\frac{\beta t}{\tau_{1}}\right)\times {\left(1+\frac{t}{t_{1}}\right)}^{\beta -2}\times exp\left[-\left(\frac{t}{\tau_{0}}\right)\times {\left(1+\frac{t}{\tau_{1}}\right)}^{\beta -1}\right]\;$$

Hence, Formula (6), also called the modified stretch exponential function (MSE), is used to interpret the relaxation data obtained by IMC. With the correction, the time derivative remains finite near zero and Formula (6) reduces to Formula (1), the KWW equation, at long time scales [17]. To obtain a summarized τ ($\tau_{MSE})$ out of $\tau_{0}$ and $\tau_{1}$, as is natively given for the KWW equation ($\tau_{KWW}$), the following formula is used [17].

(7) $$\tau_{MSE}=\tau_{0}^{\frac{1}{\beta }}\times \tau_{1}^{\frac{\beta -1}{\beta }}\;$$

Figure 4 shows the curves that are measured by IMC at different temperatures and the obtained respective $\tau^{\beta }$ values. In contrast to the DSC method, according to the KWW function, which measures the relaxation times indirectly, the rate of reaction (“the speed of relaxation”) is directly measured by IMC. This leads to manifold advantages of IMC measurement—for instance, increased resolution and exact determination of the α-relaxation times without side reactions.

### 2.4. Comparison of DSC and IMC for α-Relaxation Analysis

As the concept of α-relaxations has now been introduced, a few more terms need to be clarified, to prevent confusion. There are different definitions in the literature for “sample storage below $T_{g}$”, which would, in the case of an IMC measurement, also correspond to the measurement temperature. The terms are “annealing”, “aging”, and “tempering”. Throughout this review, the term “tempering” is used as it does not interfere with other process steps in freeze-drying. Furthermore, in practical usage and the literature, the $\tau^{\beta }$ values are almost always given instead of the single parameters τ and β because, in this way, more robust results are generated [17,53]. The possibility to investigate relaxation times with an isothermal microcalorimeter instead of using differential scanning calorimetry was first introduced by Liu et al. [17]. Since then, a few attempts have been made to compare the methods. In the following part, the measurement and evaluation methods are compared. To allow such a comparison, the values determined with DSC and the KWW function are called $\tau_{KWW}^{\beta }$, the results of IMC with the MSE function $\tau_{MSE}^{\beta }$.

#### 2.4.1. The Behavior of *τ* and *β*

Theoretically, the relaxation determination with DSC and IMC should provide the same results, but both parameters, τ and β, depend strongly on their thermal history, which includes the temperature, composition, pressure, and also the measurement method itself. It must not be forgotten that although the enthalpy relaxation ($\tau_{MSE}^{\beta }$) is considered similar to the enthalpy recovery ($\tau_{KWW}^{\beta }$), this must not always be the case [23]. Thus, practically, the estimated value of $\tau^{\beta }$ can be different between both methods. Hancock et al. investigated the relaxations of quench-cooled amorphous systems consisting of polyvinylpyrrolidone (PVP), indomethacin, or sucrose [48]. The β-values of these single-component systems correlated with the molecule size using $\tau_{KWW}^{\beta }$. Small molecules had generally higher β-values [48]. This means that small-molecule systems possess a more homogenous distribution of relaxation states compared to larger polymers. Taking into account that calorimetrically investigated α-relaxations depend on the movements of whole molecules, the observation of Hancock et al. is reasonable because small molecules are less sterically impeded than larger ones. Thus, the chance is higher that small molecules such as sucrose can quickly “move” to the same equilibrated energy state, relaxing homogenously from there on, in contrast to polymers such as PVP. Van den Mooter et al. further observed a decrease in the β-values using $\tau_{KWW}^{\beta }$ when the tempering temperature was lowered [90]. These findings were obtained during their studies on pure quench-cooled API triazolam, temazepam, and diazepam [90]. Moreover, this can be explained with molecular movement. At lower temperatures, the molecular movement of the small molecules is decreased, resulting in a prolonged time until the molecules approach an equal relaxation state, and, thus, there is a wide distribution of these relaxation substates. Bhugra et al. stated that the value of β, independent of whether it is determined as $\beta_{KWW}$ or $\beta_{MSE}$, is not coupled to temperature [53]. They support their data with correlating relaxation measurements below and above $T_{g}$ [53]. For this purpose, single-component systems of amorphous disaccharides were utilized. The obtained β-values remained similar in the applied temperature range [53]. This is the only publication that has reported such a case, which is further in contrast to the theoretical background of the α-relaxation theory. Recent studies investigated relaxation processes also by molecular modeling and tried to fix one of the parameters (τ or β) for more robust results [79]. Wilkinson et al. proposed a model where the temperature dependence of the stretch exponent β is taken into account [79]. Their results indicate an overall temperature dependency of β. It can be considered that near and around $T_{g}$, the temperature dependency of β is so small that the impact disappears in the noise of the measurement.

In general, it can be noted that the β-values themselves, as well as the combined $\tau^{\beta }$ results obtained by the MSE equation, are smaller than those measured with the KWW function [17,53]. This could be a numerical error, but it could be that the IMC method captures also some other modes of relaxation that the DSC method is not able to detect [17]. Liu et al. showed that, sometimes, the $\tau_{KWW}^{\beta }$ gives unphysical τ and β values whereas the $\tau_{MSE}^{\beta }$ gives reasonable results [17]. These measurements were performed with freeze-dried or quench-cooled pure sucrose and trehalose formulations [17]. At the moment, there is no valid explanation for this, but it is assumed that the $\tau_{KWW}^{\beta }$ tends to fail when the corresponding change in power during the IMC of the sample is very fast in the beginning [17]. As mentioned, the generation of one data point for the determination of $\tau_{KWW}^{\beta }$ needs much more effort than measuring $\tau_{MSE}^{\beta }$. The resolution in $\tau_{KWW}^{\beta }$ is strongly reduced with the consequence that, particularly at short time scales, where the relaxation occurs rapidly and strongly, the data points are not sufficient to calculate $\tau_{KWW}^{\beta }$ correctly and reproducibly. In the direct IMC measurement, a data point is created, e.g., every 2 s, whereas, in the DSC measurement, the gaps between the single points often are around 1800 s. A further advantage of determining $\tau_{MSE}^{\beta }$ is that the calculation is based on three fitting parameters ($\tau_{0},\;\tau_{1}$, and β) instead of only two (τ and β) for the $\tau_{KWW}^{\beta }$.

It should be noted that Pikal et al. developed a method to determine relaxation times only with the width of $T_{g}$ [85]. Only three publications were found that compared the$\;\tau_{∆T_{g}}^{\beta }$ with $\tau_{MSE}^{\beta }$ or $\tau_{KWW}^{\beta }$ [22,42,85]. Chieng et al. support the qualitative relationship between the KWW method and the “width of Tg method” ($∆T_{g}$), although these two methods are not fully quantitatively comparable [22,85,91]. It is concluded that the $\tau_{∆T_{g}}^{\beta }$ is qualitatively operational but more precise results can be achieved with $\tau_{KWW}^{\beta }$, or even better with $\tau_{MSE}^{\beta }$.

#### 2.4.2. General Observations for Samples with Different Thermal History

Shamblin et al. pointed out the difference between the thermal history of samples [16]. In their studies, pure substances (sorbitol, sucrose, trehalose, and indomethacin) were converted into an amorphous solid in different ways, by freeze-drying, melt-quenching, and melt-quenching with subsequent aging [16]. These systems significantly differ in their changes in heat capacity, with lyophilized systems having the highest and aged samples the lowest [16]. This means that, compared to the thermodynamic equilibrated state, which is the heat capacity above $T_{g}$, lyophilized samples also differ the most (largest $∆c_{p}$), hence having the highest energy excess among the samples. As expected, Liu et al. reported that, above $T_{g}$, the differences in heat capacity vanished and all products then had the same heat capacity [16]. To explain this, they emphasized the complex thermal history of freeze-dried samples through the stresses that affect a formulation during the process: the maximum freeze-concentrated state, followed by freezing itself and the removal of water by sublimation and evaporation [16,43]. Once again, the need for more awareness regarding thermodynamic matrix properties during the optimization of freeze-drying processes is strengthened by this point of view.

Furthermore, freeze-dried samples possess a shorter relaxation time (low value of $\tau^{\beta }$). This means that such samples are less rigid and more mobile [17,23]. Moreover, it is observed that, especially in freeze-dried samples, the relaxation energy is greater than the recovery [17]. This indicates that freeze-dried samples possess another mode of energy excess, which is not recovered at $T_{g}$ and thus not reversible, in addition to α-relaxation. Both aspects—the higher relaxation energy within shorter times and the faster molecular mobility—emphasize the fact that lyophilizates should be investigated more intensively regarding their $\tau_{MSE}^{\beta }$. A process that leads to low energy excess combined with a long relaxation time (high $\tau_{MSE}^{\beta }$ value) could strongly improve the long-term stability of freeze-dried drug products.

Obviously, the measurement via IMC is superior compared to the DSC method. It is the only possibility to truly measure the rate of α-relaxations directly. More data points can be generated easily to increase the resolution and the MSE as the fit-function leads to more reliable values of τ and β. In fact, the IMC measurement requires a greater sample mass (300 mg) compared to a single DSC measurement (10 mg), but for each data point of $\tau_{KWW}^{\beta }$, at least one measurement is needed. Even if the resolution of the $\tau_{MSE}^{\beta }$ measurement is decreased to three data points per hour in a 12 h measurement, measuring $\tau_{KWW}^{\beta }$ with the same resolution would need at least 36 DSC runs, thereby increasing the needed sample mass to 360 mg. However, it has to be kept in mind that a DSC measurement is needed to obtain $∆c_{p}$ at $T_{g}$ and the $T_{g}$ itself, to calculate $∆H_{r}\left(\infty \right)$. Furthermore, a DSC instrument is available in most laboratories that deal with freeze-drying, compared to an IMC instrument, which is rare and expensive to acquire. A summary of DSC and IMC methods in the context of relaxation determination is provided in Table A1 and Table A2, respectively.

It should be noted that the calorimetric methods also have their disadvantages compared to other relaxation determining analytics. Side effects such as crystallization or melting can superimpose relevant relaxation phenomena even when the DSC is used in modulated mode. Furthermore, methods such as dielectric spectroscopy are able to determine relaxation parameters above $T_{g}$ [53]. In IMC measurements, a high sample mass is needed and the measurement time can take even days. The resulting relaxation data of the different methods can of course differ, but, for each purpose, a certain method is more suitable. The reason that this review is strongly focused on the calorimetric methods is that it was shown in the past that the data obtained by calorimetry can be most helpful in the pharmaceutical field.

## 3. Application of *α*-Relaxation Analysis

In the following, the results and conclusions from the application of α-relaxation analysis from previously published papers are reviewed and completed by our own new data.

### 3.1. Correlation of $\tau^{\beta }$ with Storage Stability of Active Pharmaceutical Ingredients

The idea is that, with increasing relaxation time, i.e., a high value of $\tau^{\beta }$, molecular movement in the formulation is reduced. For several degradation pathways of biological entities, molecular movement is mandatory and, hence, these degradation processes should be decreased.

In 1994, Duddu et al. performed experiments to correlate the aggregation of a protein drug with α-relaxation times. For their study, lyophilized sucrose and trehalose formulations containing a chimeric monoclonal antibody in the ratio of sugar:protein 12.5:1 were utilized [92]. Although trehalose formulations have a higher $T_{g}$, the measured relaxations at 5 °C, far below *Tg*, were the same as in the sucrose formulations [92]. The mathematical reason for this is simple: the relaxations at different temperatures showed Arrhenius-like behavior for trehalose samples, while sucrose-based formulations possesses non-Arrhenius-like kinetics. Thus, the relaxation times matched at a certain temperature, around 5 °C in this case, leading to even shorter relaxation times for trehalose below this temperature. The results thus suggest that, at storage temperatures below 5 °C, the molecular mobility in trehalose-based samples is higher and degradation processes are faster than in sucrose-based glasses. With this, surprisingly, although trehalose has the higher $T_{g}$, the better stabilizer might be sucrose at the widely used storage condition of 2–8 °C. On the basis of their results, Duddu et al. normalized the “real” storage time (*t*) of their aggregation study using the relaxation time (τ) and called it the reduced relaxation time, Rrt (see Formula (8)).

(8) $$\mathrm{Reduced}\;\mathrm{relaxation}\;\mathrm{time}\;\left(\mathrm{Rrt}\right)=\frac{t}{\tau }$$

This approach tries to define the protein aggregation in relation to the “real” experiment time and the relaxation process of the respective system. Nevertheless, for both formulations, less than 2% aggregation was predicted when the reduced relaxation time $\left(\frac{t}{\tau }\right)$ was lower than 10. This means that, with the determined relaxation time ($\tau_{KWW})$ of $10^{8}$ h at 5 °C for sucrose and $10^{6}$ h for trehalose, the real storage time until 2% aggregation would occur is in the order of $10^{9}$ h and $10^{7}$ h (several years; see Formula (9)).

(9) $$\mathrm{Rrt}=\frac{t}{\tau },\;\mathrm{with}\;\tau =10^{6}\;h\;\mathrm{and}\;\mathrm{Rrt}=10\to \;t=10\times 10^{6}\;h=10^{7}\;h=1142\;\mathrm{years}$$

Although this was a well-considered work providing first impressions on the α-relaxation times of pharmaceutical products, the results question the relevance of correlating stability with α-relaxations. It appears that the differences between sucrose and trehalose formulations are so small that they do not truly matter at storage temperatures under refrigerated conditions. Furthermore, the predictive calculation in Formula (9) was not proven by real aggregation data. The authors themselves emphasized the need to perform more comparable stability studies in this area, which was done later by, e.g., Shamblin et al., in 2006. The authors were some of the first to publish an investigation on the correlation of the stability of freeze-dried formulations with α-relaxation times [93]. They investigated different lyophilized cephalosporine and ethacrynate sodium mixtures. From their results, it becomes very clear that reactions requiring whole molecule movements (such as dimerization in ethacrynate sodium) are strongly coupled with the relaxation times [93]. This is in contrast to degradation processes, which are more or less independent of molecular movement, or only need a small functional group to move (opening of a β-lactam ring in cephalosporine mixtures) and consequently show only a weak correlation to relaxation times [93]. Shamblin et al. also found out that direct formulation-specific aspects superimpose the effect of α-relaxation. Astonishingly, cefoxitin sodium showed increased stabilization of the β-lactam ring when sucrose was added, although sucrose significantly reduced the global α-relaxation time of the formulation. This occurred due to an unexpected stabilization effect of sucrose on this certain API, which showed that excipients as such can have a much stronger impact on the stability than the relaxation time. Moreover, Wang et al. reported similar results for their studies. They investigated mixtures of five different proteins in sugar matrices with different sugar/protein ratios. With the addition of sucrose to the proteins, $\tau_{MSE}^{\beta }$ increased as well as the protein stability. Up to a sugar:protein ratio of 1:1, $\tau_{MSE}^{\beta }$ correlated perfectly well with long-term stability. However, at ratios beyond 1:1, meaning with more sucrose added, $\tau_{MSE}^{\beta }$ decreased, although the protein stability from this point on further increased [94]. Quite similar studies, presenting an inconsistency between formulation parameters and relaxation, can be found in Abdul-Fattah et al., Chang et al., and Shamblin et al. [21,56,93]. Therefore, it can be concluded that α-relaxation studies are not able to directly help in the selection of the best formulation composition, but more promising results can be found for investigations concerning the connection of process parameters and relaxation.

Luthra et al. were able to detect a correlation of α-relaxation with long-term stability (rpHPLC) in mixtures of aspartam/sucrose and aspartame/trehalose [95]. In their study, subsequent to the lyophilization process, tempering was performed and the samples were kept at different temperatures below $T_{g}$ for a certain amount of time. The authors describe that tempered formulations indeed have a higher initial protein degradation compared to freshly prepared samples; however, this initial damage is (over-)compensated during storage. With initial tempering, the degradation kinetics were dramatically slowed down. Hence, tempering leads to higher sample purity in the long term despite the disadvantages at t(0) of this specially treated specimen [95]. The idea behind tempering is that, at elevated temperatures below $T_{g}$ (e.g., 60 °C for trehalose formulations), more energy excess of the sample is released in a short period of time compared to the subsequent storage conditions (e.g., 4–25 °C for pharmaceutical formulations). With this, α-relaxation time $\tau^{\beta }$ increases throughout the heating process. Hence, the molecular movement and, thus, the degradation process are slowed down. As high temperatures typically accelerate chemical degradation, the rule for tempering must be as high as necessary but as low as possible.

Abdul-Fattah et al. showed results that also emphasize the theory of tempering with moxalactam/mannitol mixtures. As a stability surrogate in their experiments, the decarboxylation rate of freeze-dried moxalactam was utilized. The results of decarboxylation and relaxation correlated over a wide range of tempering times and temperatures [31]. This means that with the increasing time and temperature of the tempering process, the decarboxylation rate was decreased. These observations suggest that if the degrading factor strongly depends on molecular motions, α-relaxations can be used as a stability predicting factor and the stability of the formulation can be increased through this tempering practice. In the most simple fashion, tempering could be conducted by the adaption of the secondary drying time and temperature. In a later study, Abdul-Fattah et al. considered this further and included vacuum-dried formulations in their studies [96]. Compared with freeze-drying and spray-drying, the vacuum-dried formulations were exposed to high temperatures at a longer time range. As expected, the relaxation recovery at $T_{g}$ of vacuum-dried samples was the highest, because they relaxed the most, as was the stability of the formulation [96].

**Figure 5:**
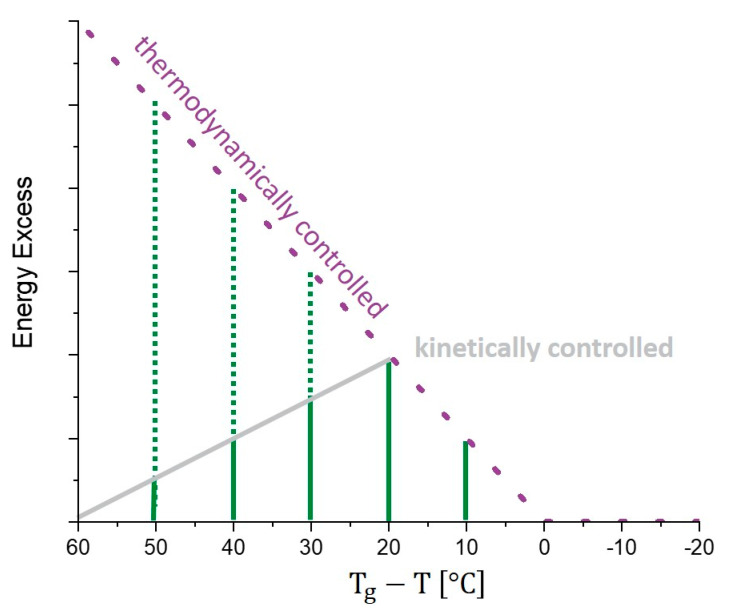
Model graph to explain the concept of an optimum tempering temperature. The purple line shows the theoretical maximum of energy excess that a sample possesses at a temperature of $T_{g}-T$ calculated with Formula (3). The grey line represents the kinetic limitation at the time scale of the experiment at the same temperature of $T_{g}-T$. The green solid lines show the measured relaxation that occurs at the time scale of the experiment, compared with the theoretical maximum, visualized by the green dotted line.

The disadvantage with tempering in all of these studies was the initial damage of the formulation. The question is whether using more moderate tempering protocols would allow an optimum to be found. Tempering at lower temperatures, perhaps with a longer tempering time, could lead to sufficient structural α-relaxations without affecting the other physical properties of the formulation [70]. Several studies conclude that α-relaxations might be negligible at 50 K below $T_{g}$ (using DSC methods) [48,90]. Thus, the optimum tempering temperature must be in the range of $T_{g}$ to $T_{g}-50$. However, the fact that different formulations result in relaxation curves with different slopes at the same $T_{g}-T$ temperature indicates that different materials react individually, with different responses to temperature variation [48]. Borde et al. suggested that enthalpy relaxation is an interaction of available thermal energy on the one side and the driving force (energetic distance toward equilibrium) on the other side [97]. At $T_{g}$, the relaxation times $\tau_{KWW}$ should be the same within different formulations, which could be proven in some studies [48]. This idea arises from the thermodynamic consideration that, at $T_{g}$, the glass reaches the supercooled, thermodynamically equilibrated, liquid state. However, Luthra et al., as well as Chung et al., found in their DSC studies that there is a maximum in relaxation not at $T=T_{g}$ but at a temperature of $T_{g}-T=20$, which is, at first glimpse, contrary to the consideration stated above. They explained the phenomenon of the mobility maximum as follows [43,84]. If the tempering happens far away from $T_{g}$ ($T_{g}-T_{a}\geq 20$), the process is under kinetic control. This means that the molecular mobility is so slow that, during the aging experiment, only a small enthalpy loss happens. When approaching $T_{g}$ ($T_{g}-T_{a}<20$), the process is under thermodynamic control. At this point, less enthalpy excess of the glass compared to the equilibrated state remains within the sample. Thus, only limited energy excess is possible and the relaxation time decreases as the tempering temperature increases [43,84]. We developed Figure 5 to summarize the important consideration of a tempering optimum. It can be seen (as well as in Figure 1a) that, at temperatures below $T_{g}$, the enthalpy excess of the glass increases with increasing distance towards $T_{g}$ compared to the thermodynamic equilibrated state. Thus, the lower the temperature is, the more energy excess is stored in the samples and the longer are the relaxation processes. When the kinetic border crosses the thermodynamic limit line, it is possible to “fully” relax a sample. From this point on, “always 100%” of the energy excess is released. Even closer to $T_{g}$, the measured enthalpy recovery decreases, because thermodynamically less energy excess is stored within the sample. This results again in a decrease in $\tau_{KWW}^{\beta }$.

Based on the existing literature, experiments were performed in our group to obtain more insights about the kinetic and thermodynamic factors of the tempering process and the relaxation behavior afterwards. As the former publications mostly use the $\tau_{KWW}^{\beta }$ method to determine the optimum tempering temperature, we decided to re-evaluate the considerations with the $\tau_{MSE}^{\beta }$ method. Directly measuring relaxation times with higher sensitivity should enable us to see whether $T_{g}-T=20\;{}^{\circ}C$ is also the optimum tempering temperature for $\tau_{MSE}^{\beta }$ and if the relaxation is in fact fully hindered at $T_{g}-T>50\;{}^{\circ}C$. Further, it was necessary to investigate whether the kinetic boarder shifts and approaches the thermodynamic limit at higher values of $T_{g}-T$ with longer time scales of the measurement, or whether, at this energy barrier, at a certain point, relaxation no longer occurs (meaning that the relaxation process slows down to a time scale of several years) although an energy excess is theoretically present. This would be relevant in the context of industrial processes where the cold chain for lyophilizates is interrupted for many days at room temperature, i.e., up to 25 °C.

**Figure 6:**
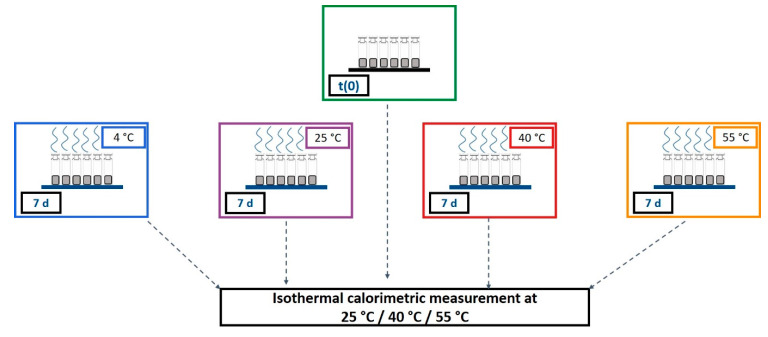
Overview on the tempering time and temperatures as well as the measurement temperatures in IMC that were used in this study.

Two placebo mixtures containing trehalose and sucrose were studied. Two freeze-drying cycles with different freezing steps were performed: one with random ice nucleation (RN) and one with controlled ice nucleation (CN). The formulations and the processes were used to cover a broad range of different $T_{g}$ values between 50 °C and 100 °C. In this study, measurement temperatures of 25 °C, 40 °C, and 55 °C were used with the IMC. Thus, measurements far from and near $T_{g}$ were possible. A tempering time of 7 days was used. Details of the composition and production of the samples can be found in Section 6, the Materials and Methods. Figure 6 illustrates the measurement procedure.

**Figure 7:**
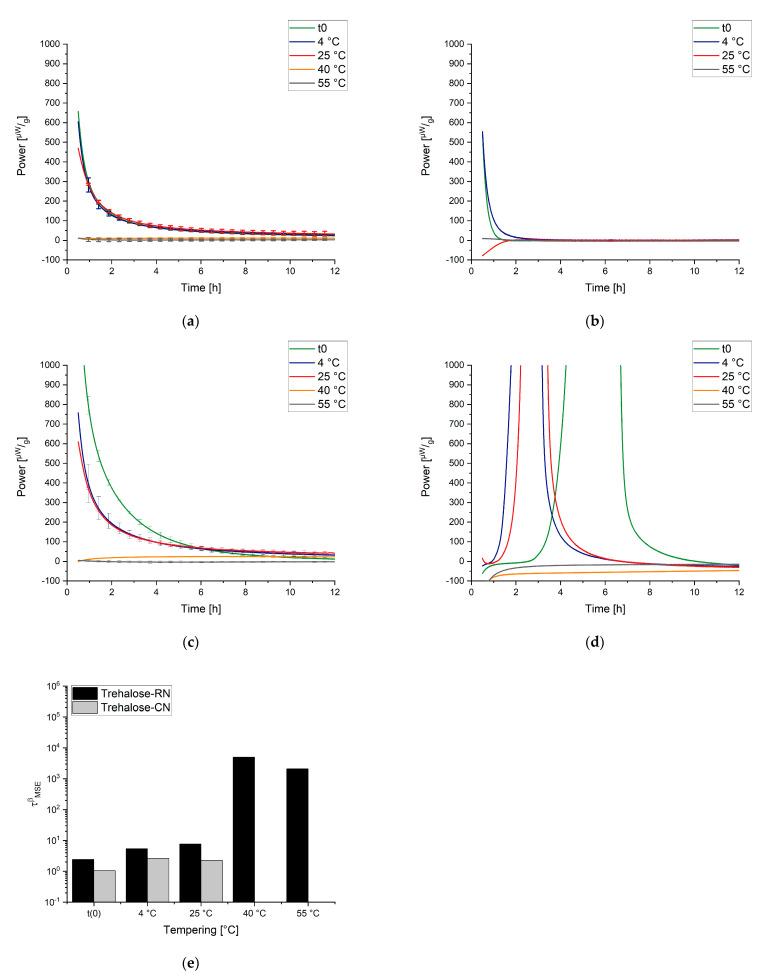
Isothermal measurements performed at 55 °C. Freshly prepared sample (green), 4 °C tempered sample (blue), 25 °C tempered sample (red), 40 °C tempered sample (orange), 55 °C tempered sample (grey). (**a**) Trehalose-RN, (**b**) Sucrose-RN, (**c**) Trehalose-CN, (**d**) Sucrose-CN, (**e**) calculated values of $\tau^{\beta }\left[h\right]$. Missing bars indicate unsuccessful curve fitting. Error bars in sucrose samples not shown for better clarity.

**Figure 8:**
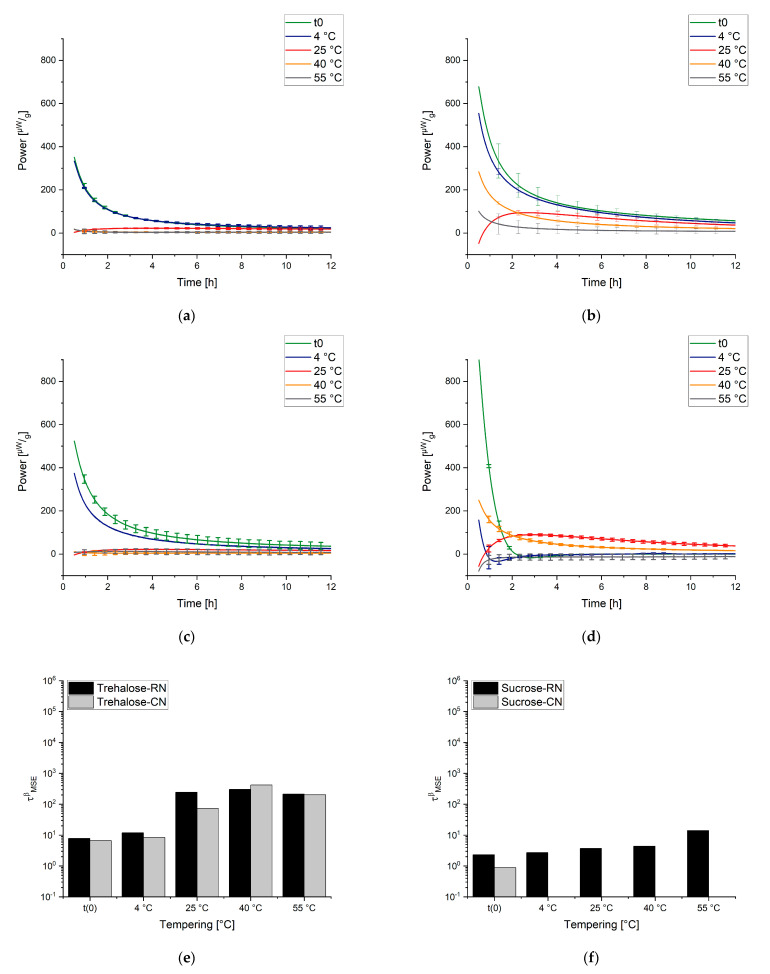
Isothermal measurements performed at 40 °C. Freshly prepared sample (green), 4 °C tempered sample (blue), 25 °C tempered sample (red), 40 °C tempered sample (orange), 55 °C tempered sample (grey). (**a**) Trehalose-RN, (**b**) Sucrose-RN, (**c**) Trehalose-CN, (**d**) Sucrose-CN, (**e**,**f**) calculated values of $\tau^{\beta }\left[h\right]$. Missing bars indicate unsuccessful curve fitting.

**Figure 9:**
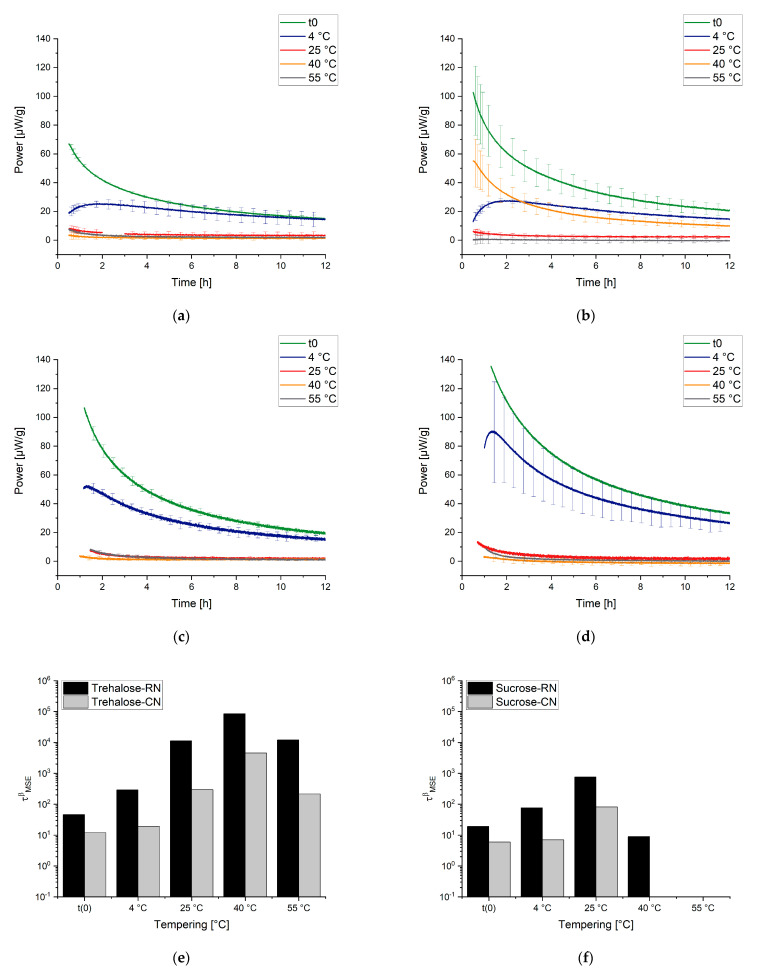
Isothermal measurements performed at 25 °C. Freshly prepared sample (green), 4 °C tempered sample (blue), 25 °C tempered sample (red), 40 °C tempered sample (orange), 55 °C tempered sample (grey). (**a**) Trehalose-RN, (**b**) Sucrose-RN, (**c**) Trehalose-CN, (**d**) Sucrose-CN, (**e**,**f**) calculated values of $\tau^{\beta }\left[h\right]$. Missing bars indicate unsuccessful curve fitting.

When the obtained results are discussed, the following has to be kept in mind. The “t(0) samples” were measured directly after the freeze-drying process; thus, the concept here is that the subsequent tempering process for the temperatures 25 °C, 40 °C, and 55 °C could be tracked in real time. IMC always measures the relaxation process while it happens, so the measurement temperature equals the tempering/storage temperature of a sample. For the samples that were tempered for 7 days at 4 °C, 25 °C, 40 °C, and 55 °C before the measurement, the tempering process belongs to the thermal history/preparation process of the sample. It was investigated how strongly the sample still relaxed after different tempering protocols. The measuring temperatures of the instruments were chosen to cover typical conditions for storage and accelerated stability studies (25 °C, 40 °C) as well as a high temperature to approach $T_{g}$ (55 °C). Much higher or lower temperatures of the instruments were not possible due to technical limitations. Figure 7, Figure 8 and Figure 9 display the resulting curves as well as the calculated $\tau_{MSE}^{\beta }$ values.

**Figure 10:**
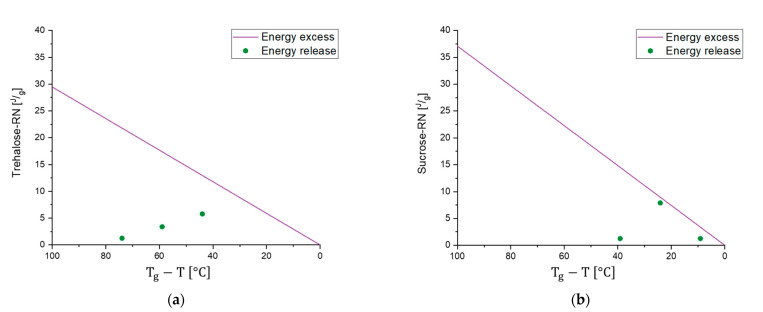
Model of Figure 5 applied to the samples Trehalose-RN (**a**) and Sucrose-RN (**b**). The purple rectangles show the theoretical maximum of energy excess that the respective sample possesses at a temperature of $T_{g}-T$ calculated with Formula (3). The green dots show the measured relaxation that occurs in the time scale of the experiment.

In the 55 °C measurement (Figure 7a), the result for the different tempering conditions of trehalose-based samples generated by a random ice nucleation process (Trehalose-RN) can be separated into two clusters, one with t(0), 4 °C, and 25 °C and the second with 40 °C and 55 °C. The cluster of t(0)-25 °C suggests that higher tempering process temperatures are needed to relax the formulation adequately. Moreover, the values of $\tau_{MSE}^{\beta }$ are low and all on a similar level (2–8 h), compared to $\tau_{MSE}^{\beta }$ for the 40 °C and 55 °C tempered samples of 5008 h and 2097 h, respectively. Consequently, 40 °C tempering over a period of 7 days seems to be the best method to increase the relaxation times in this formulation. In contrast to the findings of Luthra and Chung [41,84], however, 40 °C is not equal to $T_{g}-T=20\;{}^{\circ}C$ but rather to $T_{g}-T=60\;{}^{\circ}C$. This means that the proposed model in Figure 5 is not correct or that the intersection point of the kinetic border and the thermodynamic energy excess of trehalose formulations is not at $T_{g}-T=20\;{}^{\circ}C$. To clarify this issue, the fitted t(0) curves of Trehalose-RN and sucrose-based samples generated by the random ice nucleation process (Sucrose-RN) measured at 25 °C, 40 °C, and 55 °C were plotted in the time range of 0–12 h and then integrated. The resulting area under the curve (AUC) was converted to the unit $\frac{J}{g}$. Thus, the model in Figure 5 could be applied to the samples Trehalose-RN and Sucrose-RN. The results are displayed in Figure 10. In fact, the theoretical considerations and the model of Figure 5 seem to work very well. The AUC of Trehalose-RN increases continuously with increased temperature. With 55 °C as the highest measured temperature and a $T_{g}$ of 100 °C, all observed relaxation times were kinetically controlled. In contrast, the AUC of Sucrose-RN first increased and then decreased again at 55 °C because the intersection point of the kinetic and thermodynamic line was crossed.

The resulting curves of the trehalose-based samples generated by controlled ice nucleation (Trehalose-CN) show three clusters at the measurement temperature of 55 °C, instead of two as with the Trehalose-RN curves. Thus, it seems that 4 °C and 25 °C tempering have an impact on relaxation times compared to t(0) in Trehalose-CN samples but not in Trehalose-RN samples. Due to the different processes (RN vs. CN), both samples possess different $T_{g}$ temperatures, although they have the same excipient composition. The respective $T_{g}$ temperatures are 85 °C for Trehalose-CN and 100 °C for Trehalose-RN. These findings clearly show that the ratio between $T_{g}$ and the measurement temperature (equals storage temperature) is very important. Concerning relaxation times, this could mean that the tempering temperature is a complex issue that cannot be fixed at a specific value such as $T_{g}-T=20\;K$ and the planned storage temperature always has to be taken into account. For example, the α-relaxation times of Trehalose-RN tempered at 25 °C and 55 °C are quite similar when measured at 40 °C (Figure 8a,e) as well as when measured at 25 °C (Figure 9a,e). After tempering for 1 week at 25 °C, relaxation is the same as for tempering at 55 °C. Consequently, although kinetically limited (see Figure 5), tempering for longer times at a lower temperature can compensate for high tempering temperatures. With this consideration, tempering at 55 °C would be unnecessarily high when the samples need to be stored at 25 °C or 40 °C. In the past, tempering temperatures of 50 °C or higher were utilized [31,95]. However, the present results suggest that a tempering protocol of Storage temperature+xK is sufficient and would offer better long-term stability with less protein damage in the beginning.

With a lower measurement temperature, the absolute power signals decreased in all samples. It is noticeable that some curves show a maximum in the power signal instead of continuously approaching the x-axis. This phenomenon is called the “tempering shoulder” in the following. This tempering shoulder occurs independently of the formulation in the curves of samples that were tempered around 15–20 K below the IMC measurement temperature (e.g., in 4 °C tempered samples measured at 25 °C, 25 °C measured samples at 40 °C, and 40 °C tempered samples at 55 °C). This observation was also made by Abdul-Fattah for foam-dried formulations and they excluded this part of the curve from the calculation of $\tau_{MSE}^{\beta }$ [56]. Kawakami and Pikal stated enthalpy recovery as origin of this phenomenon [23]. They reported that the tempering shoulder cannot be easily explained by classical relaxation theory but could arise from several substates of a formulation. Whereas most parts of such a sample recover energy, there are still some small distributed substates that relax. In the beginning, thus, it is a combined peak of endothermal and exothermal events, with exothermal ones remaining in the end [23]. However, although this phenomenon is not fully explained yet, the samples that showed this tempering shoulder were also the samples with the best API stability according to Abdul-Fattah et al. [56]. The present results suggest that the obtained tempering shoulder is a phenomenon that is observable at a specific ratio of tempering and measuring temperature.

Moreover, some of the Sucrose-CN samples showed a small endothermal consumption valley after the relaxation decay. At the moment, it cannot be verified where this thermal event arises from. One idea could be that, through the controlled nucleation process, the crystallization tendency is significantly higher than in other formulations, because the residual moisture is the highest and the $T_{g}$ the lowest among the samples. Thus, this could be the consumption of a small amount of activation energy that a crystallization process needs without crystallization occurring in the timespan of the experiment.

Overall, within the same formulation, α-relaxations can be a suitable predictive tool for long-term stability, but the relevant degradation pathway must include reactions that depend on molecular movements. It has to be understood that the ingredients of the formulation always have a stronger impact on stability than relaxation. Thus, parameters such as $\tau_{MSE}^{\beta }$ are not able to improve formulation recipes but are a tool for process optimization. In the context of freeze-drying, this includes mainly the primary and secondary drying steps. The tempering of samples within a time scale of a few hours increases the long-term stability of the formulation but with slightly increased protein damage at t(0) compared to non-tempered samples. To improve the tempering procedure, experiments with IMC were conducted that showed that relaxation occurs at every temperature, even far below the $T_{g}-T=50{}^{\circ}C$ region, e.g., for samples that were tempered at 4 °C. The tempering temperature should be chosen according to the planned storage conditions. It should be good practice to temper a product at no higher than 25 °C when it is planned to be ultimately stored at 4 °C. Within a week, the kinetic limitations between samples tempered at 25 °C and 55 °C had already equalized, and with an unnecessarily high tempering temperature, more initial protein damage would occur without a significant improvement in the relaxation time.

### 3.2. Further Applications of α-Relaxation Studies

#### 3.2.1. Crystallization

The stabilization of a protein in a glassy matrix is based on the prerequisite that such an amorphous matrix is present in a sufficient quantity and does not crystallize during the relevant storage period. It is therefore highly desirable to ensure the preservation of the amorphous state and to predict how long it may take until crystallization will start. Zhou et al. found that molecular mobility can be correlated with the tendency for crystallization [98]. They investigated different small-molecule APIs and sucrose. At this point, it must be noted that the APIs were converted to the amorphous state by quench cooling, whereas sucrose was freeze-dried. Among the quench-cooled samples, mobility as well as configurational entropy correlated with crystallization tendencies [98]. To determine the crystallization tendency, DSC was utilized, applying a heating ramp up to temperatures above *T_g_* and not an isothermal approach below *T_g_*. It is suggested that crystallization above *T_g_* is probably not a good marker, because above *T_g_*, the thermal history could be erased. Surana et al. found that a longer annealing time in freeze-dried trehalose below *T_g_* lowered the onset temperature of crystallization above *T_g_* [99,100]. This result confirms that the origin of a glass (freeze-dried vs. quench-dried) has to be considered even for events above *T_g_*. Surana et al. concluded from their results that, through tempering below *T_g_*, crystallization pre-nuclei are built that have no influence on matrix mobility below *T_g_* but accelerate crystallization above *T_g_* [99]. These nuclei are not reversible and thus do not disappear above *T_g_* [99]. Later, Bhugra et al. showed that the relaxation times determined above *T_g_* with dielectric relaxation spectroscopy match the extrapolation of the relaxation times of ( $\tau_{MSE}^{\beta }$ ), determined by IMC, below *T_g_* [53]. The study was conducted with several small-molecule APIs as model compounds. This would mean that relaxation times below *T_g_* are able to govern or predict processes above *T_g_*. Bhugra et al. thus experimentally showed that coupling between *ɑ*-relaxation below *T_g_* and crystallization above *T_g_* could be plausible even without the theory of pre-nuclei. In a further study, Bhugra et al. aimed to prove this idea and investigated the onset time of the isothermal crystallization of spray-dried and freeze-dried sucrose above *T_g_* with Formula (10) [101].


(10) $$ln\left(\theta c\right)\;=\;\left(\frac{M}{\beta }\right)\times \;ln\left(\tau \right)\;+\;C$$

In the equation, *θ_c_* is the induction time of crystallization; *M* is the coupling coefficient, and *C* is a constant [101]. M has to be divided by *β* due to mathematical reasons concerning the slope of the curve by using *τ^β^* as a mobility parameter. The results showed a coupling coefficient of around 0.5, suggesting that crystallization and molecular mobility are not simply reciprocal and more factors contribute to the crystallization behavior [101]. It could additionally be noted that crystallization onset times for freeze-dried samples are much shorter than for spray-dried samples [101]. Thermodynamic driving forces, different process durations and temperatures of spray-drying vs. freeze-drying, as well as differences in surface are discussed as reasons to explain this phenomenon [101]. A coupling between relaxation times and crystallization onset below *T_g_* did not work properely [101]. Finally, reference is made to an excellent review by Bhugra and Pikal that deals with crystallization from the amorphous state, as well as to a review from Grzybowska et al. that focuses on relaxation analysis by dielectric techniques [102,103].

**Figure 11:**
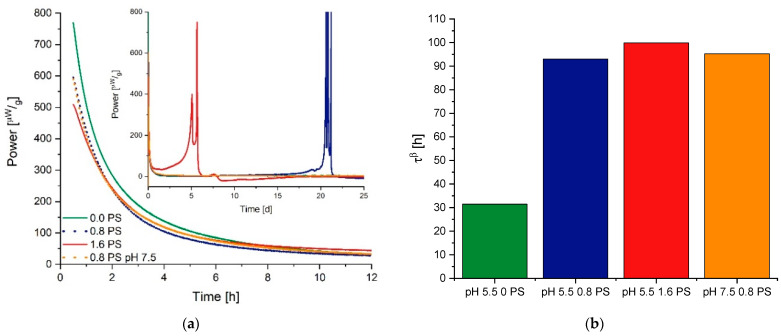
(**a**) Relaxation behavior of sucrose formulations containing 2 mg/mL IgG_1_ antibody and a different concentration of polysorbate 20 from a range 0.0–1.6 mg/mL. Except one formulation with a pH of 7.5, all other samples had a pH of 5.5. The measurement was performed by isothermal microcalorimetry with a measurement temperature of 40 °C. The small picture shows the complete measurement, which was performed over 25 days. The different crystallization peaks within a formulation most likely result from sample pooling to reach the needed amount of sample for the measurement. (**b**) Calculated values of $\tau_{MSE}^{\beta }$.

In our own set of experiments, we wished to investigate the coupling of crystallization onset with molecular mobility below *T_g_* as this is much more relevant for the storage of pharmaceutical products than the crystallization onset above *T_g_*. Our group reported in 2018 that the crystallization of sucrose in lyophilizates is accelerated by Polysorbate 20 (PS 20) [104]. To check whether this could depend on a measurable increase in molecular mobility with increasing PS 20 concentration, four different formulations containing 2 mg/mL of an IgG_1_ antibody and sucrose were investigated. They differed in the concentration of added PS 20 and/or pH. The fresh samples were measured in an isothermal microcalorimeter at 40 °C over the duration of several days. With this method, the crystallization onset of the formulation could be observed easily, in real time, and in detail. Furthermore, the relaxation as well as the onset of crystallization were recorded at once within one sample. In Figure 11, the results are presented.

It was observed that PS 20 accelerated the crystallization of sucrose, with 1.6 mg/mL PS 20 crystallizing first, followed by 0.8 mg/mL PS 20, with no crystallization during the measurement period in samples without PS 20. This was expected due to the results of Vollrath et al., 2018 [104]. However, interestingly, the values of $\tau_{MSE}^{\beta }$ increased and the curve flattened with increasing PS 20 concentration. With the knowledge that a higher concentration of PS 20 leads to an earlier onset of crystallization and the presented relaxation theory, we would have expected a decrease in the relaxation times $\tau_{MSE}^{\beta }$. Furthermore, it could be noted that the samples containing 0.8 mg/mL PS 20 possessed the same relaxation time independent of the pH value, but clearly not the same crystallization behavior. This again proves very clearly that the formulation composition has a stronger impact on the sample compared to relaxation parameters.

#### 3.2.2. Influence of the Freezing Step on *ɑ*-Relaxation

The impact of primary drying and secondary drying of a freeze-drying cycle on relaxation appears logical. Thermal treatment is executed on a forming glass by the shelves. However, the influence of the freezing step is not as clear and has not been investigated systematically so far. During freezing, a freeze concentrate is built, which itself is a glassy matrix. This glass is then dried and thus the initial glass is converted to another glass in the end.

**Figure 12:**
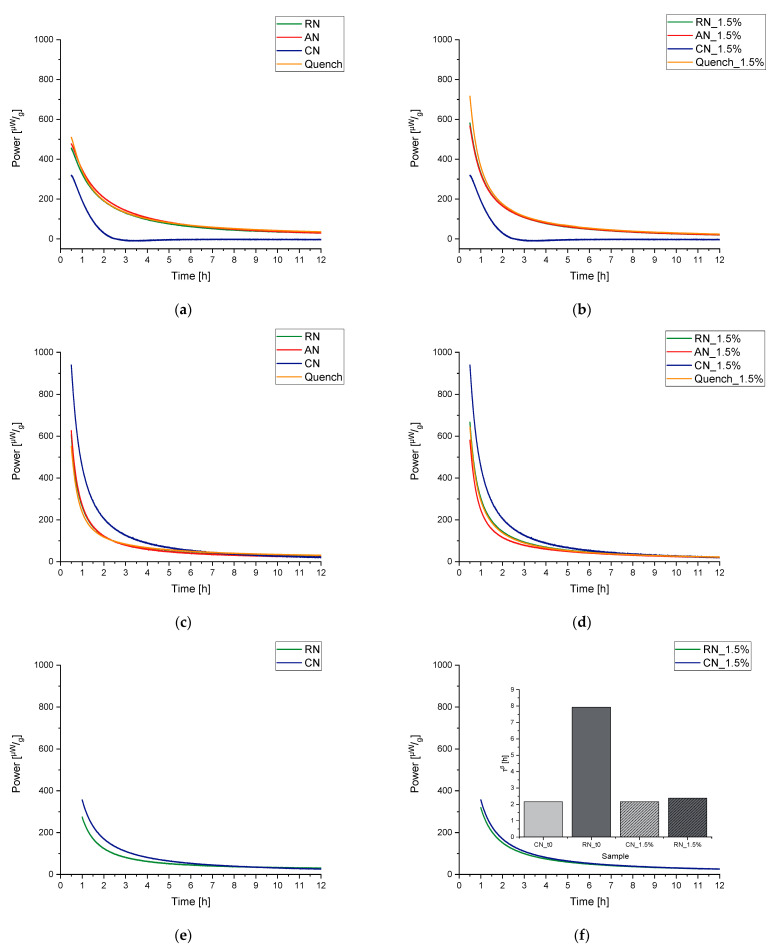
Relaxation curves of samples with different freezing processes but same compositions. (**a**,**b**) Sucrose-based formulations measured at 40 °C, (**c**,**d**) trehalose-based formulations measured at 40 °C, (**e**,**f**) trehalose-based formulations measured at 55 °C. Samples in (**a**,**c**,**e**) were measured at t(0), whereas samples in (**b**,**d**,**f**) were adjusted to the same residual moisture of around 1.5%. The small diagram in (**f**) displays the respective $\tau_{MSE}^{\beta }$ values of the corresponding samples in (**e,f**). At 55 °C, only CN and RN processes were compared.

To investigate the influence of the freezing step on the lyophilized formulation, a sucrose- as well as a trehalose-based formulation containing 2 mg/mL of an IgG_1_ antibody were prepared and freeze-dried with four different freezing processes. Random nucleation (RN), random nucleation with annealing step (AN), controlled nucleation with ice fog (CN), and quench cooling (QN) were performed and the samples subsequently equilibrated at −45 °C. Primary and secondary drying was then performed in the same freeze-drying process to ensure the same conditions. The resulting products were measured at 40 °C in the IMC, and trehalose-based formulations were additionally measured at 55 °C. The resulting data are displayed in Figure 12a,c,e. The curves of RN, AN, and QN overlap and show no significant discrepancy, while CN clearly differs. In sucrose-based formulations, samples of the CN process possess a relaxation curve with a smaller absolute heat signal and an endothermal valley after the relaxation decay, ending in a zero power signal. At both measurement temperatures, the resulting curves for the CN process of the trehalose-based samples exhibit a higher absolute power signal with stronger decay compared to the other curves.

Different freezing steps typically lead to different specific surface areas in the resulting freeze-dried cake and different levels of residual moisture. In particular, CN is used to control ice nucleation and the ice crystal size. After the water sublimates, the remaining pores in samples generated by CN are larger and the sample overall possesses a smaller specific surface area, which often leads to products with higher residual moisture [105].

With its strong influence on the glass transition itself, residual moisture clearly has a strong impact on matrix relaxations. Liu et al. found an increase in the relaxation time $\tau_{MSE}^{\beta }$ by a factor of 6 while residual moisture increased from ca. 0% to 2.7% [17]. On the other hand, Wang et al. reported that effects such as tempering overrule the influence of residual moisture [70]. For a meaningful comparison, it is therefore necessary to normalize dry matrices for residual moisture before one can determine the direct effect of CN on relaxation. Of course, the residual moisture of CN samples can be adapted to the level of the other processes by increasing the secondary drying time or temperature. However, the problem in the experimental scope is that this would cause a change in thermal history. We therefore adapted the re-moisturizing method of Lo Presti et al., to equalize the moisture level of different processes when studying the question of how nucleation affects relaxation [106]. The moisture levels were equalized to the sample with the highest residual moisture (CN samples) of ca. 1.5% and the results are presented in Figure 12b,d,f. For the ICM measurements performed at 40 °C (Figure 12b,d), the equalized moisture level led to an increase in the absolute power signal of the processes RN, AN, and QN; however, a significant difference compared to the curve of the CN sample was still observable. In contrast, when measured at 55 °C, the RN and CN curves were equal to each other, resulting in the same $\tau_{MSE}^{\beta }$ value.

Our results are consistent with those obtained by Abdul-Fattah et al. [96]. In their experiment, AN and RN samples were compared and no difference in *ɑ*-relaxation times was obtained. In trehalose-based formulations measured at 55 °C, the adjustment of the residual moisture of RN and CN samples resulted in equal relaxation behavior. Further, Chung et al. found that, in the thermodynamically controlled relaxation area (Figure 5), the slope ( $\frac{d∆H_{\infty }}{dT_{g}-T_{a}}$ ) was proportional to residual moisture [84]. However, in our case, the adaption of residual moisture should have led to the same *ɑ*-relaxation times for sucrose formulations and not for trehalose ones, as it seems to be that the former are in the thermodynamically controlled range and the latter in the kinetically controlled range (Figure 10).

All in all, the results suggest that also the freezing step itself has to be taken into account when the process parameters of freeze-dried formulations are compared. Residual moisture has an impact on the *ɑ*-relaxation times, which is explainable with the double effect of decreasing the *T_g_* temperature and simultaneously decreasing viscosity, hence increasing molecular mobility. Differences in the freezing step and residual moisture contribute separately to *ɑ*-relaxation times.

### 3.3. Collapse as a Tempering Process at Relatively Low Temperatures

**Figure 13:**
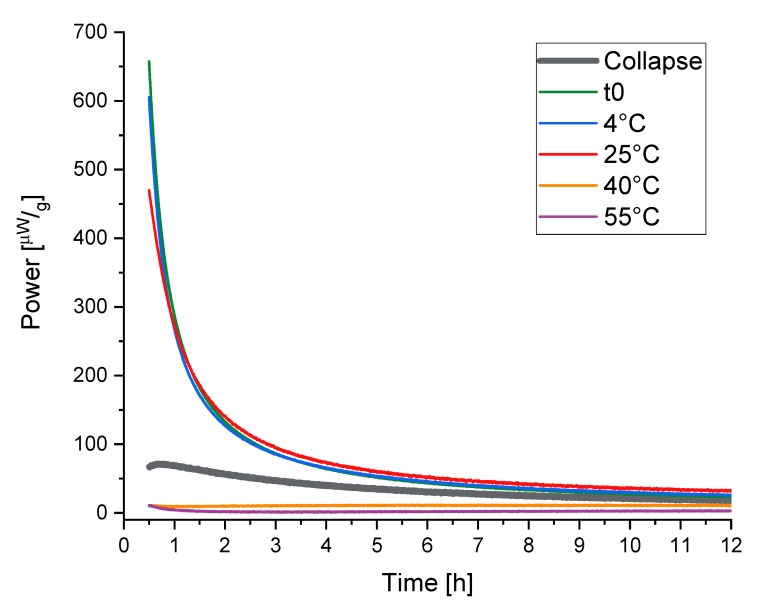
Comparison of the relaxation curve of the trehalose-based collapsed product with the curves of trehalose-based samples after conventional freeze-drying followed by tempering at different temperatures.

The basic idea is to combine the tempering process for the relaxation of the matrix within the primary drying of the freeze-drying process. Thus, one can take advantage of the relatively low $T_{g}$ of the drying matrix and the tempering process can be conducted at moderate temperatures of 25 °C. Considering full collapse drying as a method of choice, the drying is performed continuously above the $T_{g}$ of the forming glass until it reaches a high $T_{g}$, when, finally, only low residual moisture is left. As a result, the formulation does not leave the equilibrated state as it is in thermodynamic equilibrium almost until the end of the process. With this, the loss of macroscopic structure and eventually bubble formation have to be accepted. With well-conducted collapse drying, the same residual moisture as in elegantly dried cakes can be reached [30]. Moreover, in the studies of Schersch et al., protein formulations were collapsed (heated) on purpose during freeze-drying, with the outcome of improved long-term stability for collapsed protein formulations compared to elegantly dried and tempered ones [30,107]. In addition, an increased relaxation time was shown for collapsed products. Figure 13 shows how a collapsed product of a trehalose-based sample locates between freshly prepared and tempered versions. It can be considered that the tempering process of the sample happens during the collapse freeze-drying cycle and there is no need to subsequently add it afterwards. Thus, the additional heat input to relax the matrix occurs when a high amount of water is present and the absolute temperatures needed are relatively low and so is the stress for the protein. It is, on the other hand, important to restrict this period of time when a rather highly concentrated protein solution sits at a rather high temperature, as these conditions typically lead to the degradation of the protein, including aggregation.

## 4. Conclusions

Molecular mobility, especially in the form of α-relaxations, was explained, and the impact on freeze-dried products was reviewed. Although studying molecular mobility cannot primarily determine an optimal formulation recipe, it can assist in process optimization. The basis of all further considerations is the experience that the tempering of lyophilized solid products to increase the relaxation time leads to less degradation, i.e., higher storage stability. Instead of post-process tempering, we propose to use collapse or aggressive freezing drying as fast, elegant, and particularly gentle methods to remove the energy excess from freeze-dried products already during the primary drying. IMC is able to measure the heat of relaxation directly, whereas with DSC, it is only possible to measure the enthalpy recovery. Without using IMC, certain phenomena would not be detected, e.g., the tempering shoulder or the endothermal energy valley of controlled nucleated samples would be overlooked. The latter shows that controlled nucleation does more to the structure of a lyo-cake than simply increasing the ice crystal size. Furthermore, IMC leads to a better data resolution and measures modes of relaxations that are not captured with conventional DSC.

## 5. Outlook

In 2010, Wang et al. showed that, in some cases, α-relaxations are more stability determining than β-relaxations [70]. This is one of the last publications found that used IMC in the context of freeze-drying. In the last few years, although liquid protein formulations have been optimized, freeze-drying is still frequently used to produce stable dry solid protein drug products. Furthermore, new freeze-drying techniques such as controlled nucleation and collapse drying have emerged. Therefore, α-relaxation optimization by IMC and other techniques might be revisited for further improvement of product stability after lyophilization, which was neglected in the past decade.

We wish to remind the interested community of this option and we will use such α-relaxation measurements with IMC to answer the question of whether aggressive or collapse drying would be equivalent to or better for the storage stability of proteins compared to standard cycles; compared to a regular tempering practice, according to the Pikal group, IMC could further help to improve secondary drying processes in general [27,63,66,91].

## 6. Materials and Methods

### 6.1. Materials

For the preparation of placebos and monoclonal antibody formulations, the following excipients were used: IgG_1_ was in stock at Ludwig-Maximilians-Universität München (LMU), sucrose was obtained from Sigma-Aldrich (Steinheim, Germany), D(+)-Trehalose dihydrate was purchased from VWR chemicals (Leuven, Belgium), L-Methionine, U.S.P. was used from J.T. Baker (Center Valley, Pennsylvania), Histidine from Alfa Aesar (Kandel, Germany) was utilized, and Polysorbate 20 was provided by Croda (Nettetal, Germany).

### 6.2. Preparation of Formulations

The composition of the formulations that were used in the performed experiments can be found in Table 1. 

### 6.3. Freeze-Drying

For the elegant cakes, the primary and secondary drying step were kept the same using a Christ ε2-6D laboratory scale freeze-dryer (Martin Christ, Osterode am Harz, Germany). Primary drying was performed at a pressure of 0.09 mbar and a shelf temperature of −25 °C. Secondary drying was performed at 0.09 mbar applying a 0.15 K/min temperature ramp to 30 °C, which was then held for 4 h. After the drying, samples were immediately cooled to −45 °C and stoppered under a nitrogen atmosphere.

Random nucleated freezing (RN) was performed with equilibration at 5 °C for 1 h followed by ramping down to a setpoint of −45 °C with a cooling rate of 1 K/min. The temperature was then held constant for 2.5 h.

Annealing was performed with the freezing step of RN but followed by a subsequent temperature increase to −20 °C with 1 K/min and a hold time of 1.5 h. Afterwards, the samples were again cooled down to −45 °C with a rate of 1 K/min and an additional hold time of 1.5 h before the primary drying time was started.

Freezing with controlled nucleation (CN) additionally included a further equilibration step at −5 °C for 1 h followed by the introduction of ice crystals using a LyoCoN system (Martin Christ, Osterode am Harz, Germany).

Quench cooling was performed by purging the sample tray with liquid nitrogen. After 2 min, the samples were placed in the freeze-dryer, which was precooled to −45 °C. The samples were then held for 1.5 h at −45 °C before primary drying was conducted.

The freezing step of collapse intended freeze-drying was performed as for randomly nucleated samples. The mixed primary and secondary drying step begun with a pressure of 2 mBar and a temperature ramp from −45 °C to 45 °C with a heating rate of 0.7 K/min. The conditions were then held for 24 h. The pressure was then decreased to 0.03 mbar and again held at 45 °C for 20 h. After the drying, samples were immediately cooled to −45 °C and stoppered under a nitrogen atmosphere.

### 6.4. Sample Tempering

Sample tempering was performed after the freeze-drying process with crimped and closed samples. Drying cabinets were set to the chosen temperatures and the tempering duration was 7 days.

### 6.5. Differential Scanning Calorimetry

A Mettler Toledo DSC 821e (Gießen, Germany) was used for DSC experiments. For the samples in Figure 2a, a linear measurement method was used. At first, the sample was cooled and held at −10 °C for 3 min. Subsequently, the first heating cycle was performed with a heating rate of 10 K/min up to 140 °C. Then, the sample was again cooled to −10 °C with a cooling rate of −10 K/min and held at this temperature for 3 min. The second heating cycle was performed similarly to the first heating cycle. Only the two heating cycles are shown in the diagram. For mDSC, a temperature range of −10–180 °C was measured with a heating ramp of 2 K/min, an amplitude of 1 °C, and a period of 120 s. A collection of further DSC methods from the literature can be found in Table A1.

### 6.6. Isothermal Microcalorimetry

A LKB 2277 Thermal Activity Monitor (TAM) (ThermoMetric AB, Järfälla, Sweden) equipped with 4 mL ampoule twin cylinders (2277-201) was used to determine the isothermal heatflow. The instrument was electrically calibrated in the 300 µW area as proposed by Shamblin et al., and the function verified with the suggested chemical methods from Beezer et al. [93,108,109]. We experimentally demonstrated that breaking the lyophilized cake into powder and its transfer into the stainless steel ampoules does not disturb the relaxation measurement. This was performed by generating lyophilized samples directly in glass measurement ampoules with subsequent measurement without mechanically dividing the sample (data not shown). However, as presented in the technical note, rubber stoppers on glass vials can generate a significant interfering signal [110]. Thus, stainless steel ampoules were used for further measurements.

The freeze-dried samples were transferred to stainless steel measuring ampoules in a dry air-purged atmosphere. Approximately 150 mg sample was used (if necessary, pooled from several product vials). The reference ampoule stayed empty (in contrast to using crystalline glycine as in reference [17]). The measurement and reference ampoule were first lowered to the thermal equilibrium position and held for 15 min to allow equilibration (in contrast to 30 min [17]). Both ampoules were then slowly lowered to the measurement position of the TAM. The experiment was performed at 25 °C, 40 °C, and 55 °C, respectively. Data points were collected in intervals of 2 s for the first hour of the measurement and afterwards with 10 s intervals. The duration of the measurement was 12–24 h. In the literature, often, measurement times of 48 h or even more are used [17,21] but the shape of Figure 4 suggests that the time of the measurement can be strongly reduced to increase the performance of IMC measurements. In particular, the curve determined at a temperature of 55 °C shows that even after 2 h more, the heat power signal is reduced by more than half. This should be adequate for a reproducible fit. To prove the considerations, samples of Trehalose-RN and Sucrose-RN were measured for more than 100 h at 25 °C and different time intervals were used for the fit. Table 2 displays the results. The determined values of $\tau_{MSE}^{\beta }$ did not change if the fit time exceeded 25 h, or, for Su-PlaM, even 12 h. Of course, a standard procedure cannot be provided here. Variables such as sample formulation, freeze-drying process, and temperature of the calorimeter have to be taken into account. For example, at a measurement temperature of 55 °C, the evaluation time can be even reduced to 12 h (data not shown). It is important to bear in mind that the start of the fit begins at 0.5 h at the earliest, due to the noise of friction through sample insertion in the instrument. A collection of other IMC methods from the literature can be found in Table A2 of Appendix B.

### 6.7. Curve Fitting

The software OriginLab 2019b was used to perform the non-linear curve fitting. An iterative algorithm based on Levenberg–Marquardt was utilized. The start values were chosen as 2 for $\tau_{0}$, 1 for $\tau_{1}$, and 0.1 for β. To verify the function of the fit, literature data were plotted, where possible, and the plotted curve refitted with the settings in OriginLab. The obtained $\tau_{MSE}^{\beta }$ values were consistent with the presented data from the literature, e.g., [23,30].

### 6.8. Karl Fischer Titration

For the determination of residual moisture content, Karl Fischer titration was utilized. Approximately 20 mg of sample was transferred into empty 2R vials in a glove box with humidity lower than 10%. A coulometric Karl Fischer titrator Aqua 40.00 (Elektrochemie Halle, Halle, Germany) equipped with an external oven was used. The oven was set at 100 °C to extract the water from the sample. The extracted water was then transported into the calorimetric cell.

## Figures and Tables

**Table 1:** Compositions of formulations used in the different experiments.

$\mathrm{Substance}\;[\frac{g}{l}]$	Sucrose-Placebo	Trehalose-Placebo	Su5.5-2-0PS	Su5.5-2-8PS	Su5.5-2-16PS	Su7.5-2-8PS	Tr.5.5-2-8PS
IgG_1_	-	-	2	2	2	2	2
Sucrose	79.45	-	79.45	79.45	79.45	79.45	-
Trehalose	-	79.45	-	-	-	-	79.45
Methionine	1.5	1.5	-	-	-	-	-
Histidine	0.42	0.42	0.42	0.42	0.42	0.42	0.42
Polysorbate 20	0.4	0.4	-	0.8	1.6	0.8	0.8

**Table 2:** Comparison of the calculated $\tau_{MSE}^{\beta }$ values from the same 100 h measurement. End times were chosen between 12 h and 100 h.

Fit time Onset [h]—Endset [h]	$\mathrm{Sucrose}\;\mathrm{Formulation}\;\tau_{MSE}^{\beta }\left[h\right]$	$\mathrm{Trehalose}\;\mathrm{Formulation}\;\tau_{MSE}^{\beta }\left[h\right]$
0.5–12	11	41
0.5–25	11	37
0.5–50	11	37
0.5–100	11	37

## Data Availability

Data are contained within the article.

## Appendix A

**Table A1:** Collection of DSC methods as suggested by different authors. n.a., not available.

**Author**	Purpose	Sample Weight [mg]	Lid	Heating Rates [K/min]	Hold Temperatures above *T_g_* [K]	Tempering Temperature below *T_g_* [K]
Hancock et al. 1995. [48]	Investigate relaxation times of pure, melt-quenched substances with DSC	2–10	Pierced	20	10–25	16–47
Van den Mooter et al. [90]	Investigate relaxation times of pure, melt-quenched APIs with DSC	2–6	Closed	10	20	16–66
Shamblin et al. [16]	Temperature + enthalpy of fusion of lyophilized substances	±5	Closed	10	n.a.	n.a.
	$c_{p}$ values of lyophilized substances	±5	n.a.	2 * (60, 0.5)	n.a.	n.a.
	Relaxation times of pure lyophilized substances with mDSC	±5	n.a.	n.a.	15	15
Aso et al. [111]	Correlation of mobile mobility and crystallization of quench-cooled samples	±5	Pierced	20	45	25
Liu et al. [17]	Investigation and comparison of relaxation times of quench-cooled and freeze-dried samples. Additionally to DSC, also IMC was used for determination	5–10	Closed	n.a.	n.a.	n.a.
Zhou et al. [98]	$c_{p}$ values of lyophilized and quench-cooled substances	±10	Closed	1 * (100, 0.5)	50	20–50
Surana et al. [99,100]	Investigate relaxation times and crystallinity onset of freeze-dried trehalose	4–5	Pierced	10	n.a.	20–60
Chang et al. [21]	$T_{g}$$\;\mathrm{and}\;∆c_{p}$ determination for IMC	5–10	Closed	1 * (100, 0.5)	n.a.	n.a.
Luthra et al. [41]	Enthalpy recovery and *T_g_* mDSC were used, where enthalpy recovery might be superimposed by *T_g_*	5–10	Closed	10	25	50-15
				1 * (80, 0.85)		
Wang et al. [70]	$T_{g}$$\;\mathrm{and}\;∆c_{p}$ determination for IMC	n.a.	n.a.	2 * (120, 1)	30	n.a.

* mDSC (period time [s], amplitude [±K].

## Appendix B

**Table A2:** Collection of performed relaxation measurements by different authors.

Author	$\tau^{\beta }\;$Method	Formulation	Outcome
Abdul-Fattah et al. [31]	MSE	Freeze-dried moxalactam mannitol system	Tempering (called annealing in the original literature) increased the structural relaxation time $\tau_{MSE}^{\beta }$ of the samples, leading to improved long-term stability.
Abdul-Fattah et al. [56]	MSE	Mixtures of monoclonal antibodies with increasing sucrose content prepared by freeze-drying, spray-drying, and vacuum drying	“Initial endothermic response” in sucrose-rich vacuum-dried formulations appeared with unclear origin. With increased sucrose fraction, stability of the antibodies increased. Correlation between relaxation times and protein depending of the process was better for fast dynamics (not the topic of this review) than for global *α*-relaxation times.
Bhugra et al. [53]	KWW and MSE	Pure quench-cooled systems of indomethacin, nifedipin, ketoconazol and flopropione	Relaxation time constants above and below *T_g_* are highly correlated.
Duddu et al. [92]	KWW	Trehalose- or sucrose-based formulations of a monoclonal antibody	Trehalose-based formulations showed Arrhenius-like behavior while sucrose showed non-Arrhenius kinetics. Thus, although sucrose is in general the more fragile glass, at temperatures below 5 °C, trehalose seems to be more fragile.
Hancock et al. [48]	KWW	Pure quench-cooled systems of indomethacin, PVP, and sucrose	Relaxation time at $T_{g}-50\;{}^{\circ}C$ is up to many years. Relaxations could predict stability of some pharmaceutical products.
Liu et al. [17]	KWW and MSE	Pure quench-cooled or freeze-dried systems of saccharides (sucrose, trehalose, raffinose, lactose, stachyose)	$\tau_{MSE}^{\beta }$ is obtained with less labor, more data points than $\tau_{KWW}^{\beta }$$.\;\tau_{MSE}^{\beta }$ is furthermore precise and enables the measurement of samples that relax very slowly with only a minor release of heat. Further, a rational development of peptide and protein formulations should be possible.
Kawakami and Pikal [23]	KWW and MSE	Review	The evaluation with $\tau_{MSE}^{\beta }$ is superior compared to $\tau_{KWW}^{\beta }$. In general, $\tau^{\beta }$ is suggested as parameter of choice instead of comparing the single parameters *τ* and *β*, respectively.
Shamblin et al. [93]	MSE	Freeze-dried amorphous drug mixtures	The correlation of *α*-relaxation with protein stability is dependent on the rate controlling step of the degradation process. The latter should be coupled to molecular mobility as well as *α*-relaxation.
Luthra et al. [41,95]	KWW and MSE	Freeze dries sucrose/aspartam and trehalose/aspartame formulations	Tempering (called annealing in the original study) significantly decreases the degradation rate and an optimum temperature for the tempering process was set $T_{g}-T=20\;{}^{\circ}C$.
Van den Mooter et al. [90]	KWW	Pure quench-cooled systems of diazepam, temazepam, and triazolam	Molecular mobility might be unimportant for temper-atures less than $T_{g}-50\;{}^{\circ}C$. Relaxations could predict stability of some pharmaceutical products.
Wang et al. [94]	KWW and MSE	Sucrose:protein mixtures in different ratios	MSE correlates with protein stability up to a su-crose/protein mass ratio of 1:1 (increase in $\tau_{MSE}^{\beta }$) well as FTIR. With higher sucrose concentration be-yond 1:1, $\tau_{MSE}^{\beta }$ decreases although protein stability further increases; thus, $\tau_{MSE}^{\beta }$ did not correlate at this ratio anymore.
